# Short and long-term clinical effectiveness and cost-effectiveness of a late-phase community-based balance and gait exercise program following hip fracture. The EVA-Hip Randomised Controlled Trial

**DOI:** 10.1371/journal.pone.0224971

**Published:** 2019-11-18

**Authors:** Kristin Taraldsen, Pernille Thingstad, Øystein Døhl, Turid Follestad, Jorunn L. Helbostad, Sarah E. Lamb, Ingvild Saltvedt, Olav Sletvold, Vidar Halsteinli

**Affiliations:** 1 Department of Neuromedicine and Movement Science, NTNU, Faculty of Medicine and Health Sciences, Trondheim, Norway; 2 Trondheim Municipality, Trondheim, Norway; 3 Department of Public Health and Nursing, Faculty of Medicine and Health Science, NTNU, Trondheim, Norway; 4 Oxford University, Oxford, United Kingdom; 5 Department of Geriatrics, St.Olavs University Hospital, Trondheim, Norway; 6 Regional Center for Health Care Improvement, St. Olavs Hospital, Trondheim University Hospital, Trondheim, Norway; Universitat de Valencia, SPAIN

## Abstract

The aim of this trial was to evaluate the clinical effectiveness and cost-effectiveness of a home-based exercise program delivered four months following hip-fracture surgery. In the two-armed randomized, single blinded clinical trial we included persons who lived in the catchment area, were 70 years or older, and community-dwelling at time of the fracture. We excluded persons who were unable to walk ten meters prior to the fracture, and those who were bedridden or had medical contraindications for exercise at baseline (ie. four months after the fracture). All participants underwent routine treatment and rehabilitation. The intervention group received additional 20 sessions (10 weeks) structured, home exercise targeting gait and balance, delivered by physiotherapists in primary health care. Gait speed was the primary outcome. Secondary outcomes included physical activity, gait characteristics, cognitive function, activities of daily living, health-related quality of life, and health care costs extracted from hospital and municipality records. In total, 223 participants were included. Four months post surgery 143 were randomized for the exercise trial (70% women, mean age 83.4 (SD 6.1) years, mean gait speed 0.6 (SD 0.2) m/sec). Estimated between group difference in gait speed was 0.09 m/sec (95% CI: 0.04 to 0.14, p<0.001) at posttest and 0.07 m/sec (95% CI: 0.02 to 0.12, p = 0.009) 12 months post surgery. The mean between-group QALY difference was -0.009 (95% CI: -0.061 to 0.038). The mean between-group total cost difference was +242.9 EUR (95% CI: -8397 to 8584). Our findings suggest that gait recovery after hip fracture can be improved by introducing a home-based balance and gait exercise program four months post surgery, without increasing total health care costs. Future research should focus on how to implement gait and balance exercise in comprehensive interventions that increase adherence among the most vulnerable persons and have an effect on daily life activities and patient-centred outcomes.

**Trial registration:** ClinicalTrials.gov NCT01379456.

## Introduction

Hip fracture in old age represents a dramatic change in life situation [1, 2] and triggers increased use of health and care services [3, 4]. Despite recent advances in peri-operative care, hip fractures are still associated with severe decline in gait and mobility, increased risk of disability, new falls, dependency, admittance to nursing home, and excessive mortality up to ten years following the fracture [5, 6]. Recovery of pre-fracture function is less likely after hip fracture than for other fall-related injuries [7], and rate of recovery is slower in people with impaired pre-fracture function [8, 9]. Slow recovery and poor outcomes are probably related to high prevalence of frailty in older people experiencing hip fractures, which is important to consider when designing rehabilitation programs [10, 11].

Gait speed is regarded as a vital sign and a robust indicator of health and function in older adults [12] and is recommended to be used as an outcome in clinical trials including frail populations [13]. Slow gait speed is closely associated with dependency in activities of daily living (ADL) and is a strong predictor for future adverse health outcomes [14]. Older people who have sustained a hip fracture report mobility to be the most valued outcome of rehabilitation [15]. Gait function stabilizes around one year following hip fracture [16], which is far beyond the point when formal rehabilitation usually ends. Consequently, it is possible that frail older persons who sustain a hip fracture do not get the opportunity to fulfill their rehabilitation potential within the frames of standard rehabilitation. Systematic reviews conclude that extended rehabilitation following hip fracture, delivered outside a hospital setting and after formal rehabilitation is completed, has a beneficial effect on gait and mobility [17, 18]. Safe and efficient gait is a prerequisite for independent living, but few exercise trials in hip fracture patients have specifically targeted gait or included outcomes on gait control beyond speed. Structured exercise has been shown to improve physical function when delivered in a home setting with minimal supervision [19] or as progressive strength training at an outpatient clinic [20–22]. Cognitive impairment [23], depressive symptoms [24, 25], and limitations in outdoor mobility [26] are common among older people with hip-fractures and may be a barrier for participation in clinical trials. At present, increased costs are often an argument used against supervised, homebased exercise [27], however, the cost-effectiveness of such interventions has been scarcely evaluated.

### Objectives

The aim was to evaluate the clinical effectiveness and cost-effectiveness of offering a 10 weeks, home-based, structured exercise program, targeting balance and gait, four months after hip fracture, as compared to routine follow-up of community-dwelling older persons after hip fracture. We hypothesized that this exercise program would improve gait efficiency and speed and be beneficial for overall health as indicated by reduced health care costs.

## Methods

### Trial design

This was a two-armed pragmatic, stratified and randomized controlled trial (RCT) with blinded assessors, performed at a hospital in central Norway from February 2011 to March 2014. The EVA-hip protocol and intervention have been published previously [27]. Screening for eligibility was performed, during the hospital stay and. information on pre-fracture function collected within five days after the surgery (T0) either at the hospital or by telephone if discharged from hospital. After four months (T1) participants were invited for baseline testing and a medical examination by geriatrician. Those who met the inclusion criteria were then randomized to an intervention or a control group. Study-related assessments were performed at completion of the intervention two months (T2) and after eight months (T3) after randomization. Assessments were performed in a movement laboratory at the University Hospital. Organized transport was offered, and participants unable or reluctant to attend were assessed at home with a modified protocol. Participants were instructed not to provide information that could reveal group allocation to the researchers and assessors, and this information was repeated prior to each assessment. Blinding of physiotherapists delivering the intervention was not possible.

Patients or their next-of-kin gave written informed consent at T0 and all participants confirmed their consent at T1 before randomization. The study was approved by the Regional Committee of Ethics in Medical Research (REK 2010/3265-3, 24.01.2011).

The authors confirm that all ongoing and related trials for this intervention have been registered. The date of first participant enrolment was in February 2011, this was the start of screening of potential participants from operation lists. Our trial registry was submitted on June 23, 2011, before the first randomisation of participants to the exercise intervention four months later. This two-step procedure allowed us to describe what characterized those able or willing to participate in the exercise intervention.

### Participants

Eligible participants were community-dwelling in Trondheim municipality prior to the fracture, 70 years or older, diagnosed and operated for intra-capsular or extra-capsular hip fractures (International Classification of Diseases ICD-10 S72.0-S72.2), and identified by experienced physiotherapists by use of hospital admission lists. At T0 exclusion criteria were pathological fracture, less than 3-months life expectancy, inability to walk 10 m (with or without walking aids) before the fracture, or participating in conflicting research projects. At T1 participants were excluded after a medical examination if they had contraindications for training (unstable medical conditions) or were bedridden.

### Intervention

All participants received rehabilitation and health care services according to usual practice, varying from no follow-up at all to quite extensive rehabilitation. In addition, participants in the intervention group received two exercise sessions a week for ten-weeks, starting four months post-surgery. The intervention was delivered in participants’ homes by ten physiotherapists representing a span in experience and competence from one to 40 years of clinical work experience. They were given a short introduction to the intervention program, and had written material describing the exercises, levels of progression, and routines for how to prescribe each exercise session available.

The program was targeting balance and gait and consisted of five individually-tailored weight-bearing exercises, all entailing change in base of support: walking, stepping in a grid pattern, stepping up on a box, sit-to-stand, and lunge. Each exercise was described at five levels with increasing challenge (i.e., increasing speed, more challenging stepping and gait tasks, and increasing demands for divided attention by adding secondary cognitive tasks). Compensating strategies such as hand support or asymmetric weight bearing were kept to a minimum. Starting levels and when to progress to the next levels were decided on an individual basis by the physiotherapists. Details of the program are described in the protocol paper [27].

### Outcome measures

The primary outcome measure was **gait speed** recorded by an electronic walkway (GAITRite®), where participants were instructed to walk at a preferred speed. For participants tested in their home or not able to complete the full walk protocol, gait speed from a 4-meter walk test was used [28].

Secondary outcome measures included: temporal-spatial **gait variables** derived from the GAITRite® mat [29]. The reported variables were chosen based on earlier work in hip fracture patients, using a factor analysis approach [30]. Step length (SL), cadence, walk ratio (ratio SL/cadence), double support time, single support asymmetry, step width and step length variability (SD) were calculated as the mean of two walks at preferred speed. **Physical activity** was measured continuously over four days by a single-axis accelerometer (activPALs from PAL Technologies ltd, Glasgow, UK), attached to participant’s non-affected thigh [31], with mean upright time (standing and walking) and mean number of upright events (sit-to-stand transitions) per day (24 hours) as outcomes. **Mobility** was assessed by the Short Physical Performance Battery (SPPB) [28], and **Basic and instrumental ADL** (I-ADL) by the Barthel Index [32] and the Nottingham Extended I-ADL Scale [33]. **Cognitive function** was evaluated by the Mini- Mental State Examination [34] and the Clinical Dementia Rating (CDR) Scale [35], **depression** by the Geriatric Depression Scale [36], **and health-related quality of life** by the EuroQol-5 dimension-3L (EQ-5D-3L). The different health states generated from the EQ-5D-3L were assigned values from the UK time-trade-off tariff [37] (ie, each health state was assigned a number between -0.594 and 1.000). **Falls efficacy** was measured by the 7-item Short Efficacy Scale International (FESI) [38], and **chronic fatigue** by the Chalder Fatigue Questionnaire [39], scored on a Likert scale (0–3) providing a total score ranging from 0–33. Number of new falls during the 12 months follow-up period was registered based on retrospective reports at T3.

We assessed cost-effectiveness from a broad health care perspective. Patient utilization of primary care and hospital services were collected from local and national registers and combined with unit costs to calculate cost per patient. Costs were measured in 2012 euros (EUR) and calculated for the physiotherapy, home-based services, nursing home stays, general practitioner visits and hospital services (see S5, S6 and S7 Tables for details).

### Adverse events

Adverse events were defined as any undesirable experience during the intervention and follow-up period reported to the monitoring committee by the physiotherapists responsible for the research intervention. A medical doctor determined the relatedness to the intervention of the events reported.

### Sample size

The study was designed to have 90% power to detect a clinical meaningful difference of 0.15 m/sec in gait speed 12 months following the fracture, using a two-sample t-test with a significance level of 0.05. Assuming 15% mortality rate, 40% loss to follow-up, and a SD of 0.23 for gait speed based on earlier work including the same population [40], we calculated that 220 participants were needed for inclusion at T0 to provide n = 54 in each arm at T3. In the statistical analysis, the test for group differences in gait speed was performed as Wald tests, within the framework of a linear mixed model. The empirical SD for gait speed in the two arms and at the three time points (T1, T2, T3) turned out to be in the range from 0.21 m/sec to 0.25 m/sec, while the mortality rate and loss to follow-up were 10% and 34%, respectively.

### Randomisation

Participants were randomly allocated to the exercise or control groups after T1 by permuted block randomization, stratified by type of fracture (intra/extracapsular) and pre-fracture rollator use indoor (yes/no). The randomization was performed using a web-based randomization system developed and administered by the Unit for Applied Clinical Research, Norwegian University of Science and Technology, where the block sizes were determined by the program during the randomization process (5+5 in the first block, 1+1 in the other blocks).

### Blinding

Assessors were blinded to group allocation. The randomization was managed by a person not involved in delivery of the intervention or study-related assessments. Blinding of participants was not possible.

### Statistical analysis

All randomly assigned participants who met the inclusion criteria were included in the analysis (n = 143) [41].

The data were summarized as mean and SD or median and Inter quartile range (IQR) for continuous data and counts and percentages for categorical data.

The intervention effects for primary as well as secondary outcome variables and costs were analyzed using linear mixed models (LMMs), including the factors time and intervention and their interaction, adjusting for age, sex, and the stratification variables type of fracture (intra-/extracapsular) and pre-fracture rollator use. The LMM was specified such that the estimated means were constrained to be equal at baseline (T1) as participants were randomly allocated to the two study arms [42]. A random, subject-specific intercept was used to account for within-subject correlations, implying a compound symmetry correlation structure. Likelihood ratio tests were used to assess overall intervention effects and post hoc pairwise comparisons for time and intervention effects were carried out by Wald tests based on the estimated regression coefficients. Normality of raw data and residuals were assessed by Anderson-Darling tests and visual inspection of normal quantile-quantile plots. In the case of departures from the normal distribution the data were log- or square-root transformed if suitable, otherwise appropriate non-parametric tests were used. The results are presented on original scales, after back transforming the results obtained from transformed data. Back-transformed confidence intervals (CIs) were obtained by stochastic simulation, assuming a joint normal distribution for the estimated regression coefficients and inserting the mean values for the additional covariates. Estimated differences and CIs were obtained by bootstrapping with 10000 samples when non-parametric tests were used. Two-sided p-values <0.05 were considered statistically significant. No formal adjustment for multiple testing was included.

We calculated QALYs with an area-under-the curve approach, with the assumption of piecewise linear change in EQ-5D-3L values over time. Cost-effectiveness was evaluated by calculating the incremental cost-effectiveness ratio (ICER), that is the difference in mean costs divided by the difference in mean QALYs for the period T1-T3. For the cost-effectiveness evaluation missing data were imputed by multiple imputation (MI) using MI by chained equations, as implemented in the mice package in R [43]. The uncertainty of the ICER was assessed by bootstrapping, using 1000 bootstrap samples from the original data set (including the missing values) and performing MI for each bootstrap sample [44].

The analyses were carried out using the IBM Statistics SPSS 23 software and the R statistical package [45].

## Results

### Recruitment and adherence

**Fig 1:**
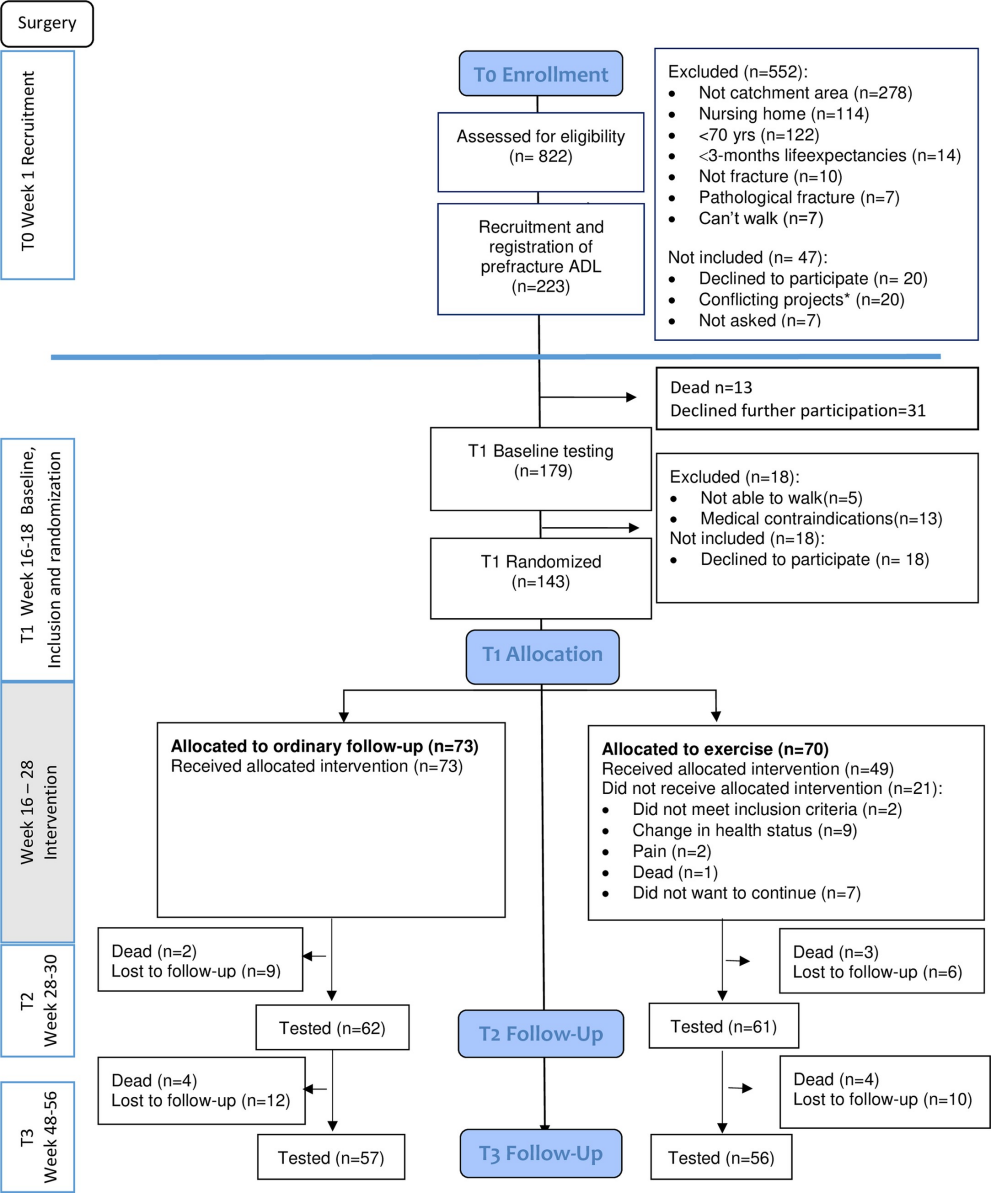
Flow of participants in the study. Flow of participants in the study showing number of participants who were assessed at each follow-up, T0—T3. All randomised participants are included in the analysis (n = 143). Recruitment and inclusion were performed in two steps. *Twenty participants were admitted for a second hip fracture within two years and were already participants in a study on orthogeriatric care.

By screening of admission lists we identified 250 persons who fulfilled the eligibility criteria, of whom 223 were included at T0. Participants flow through each phase of the study, is presented in Fig 1. Following baseline testing and the medical examination at T1 we randomized 143 participants. Participants who were included at T0 but not randomized at T1, scored lower on measures of ADL and cognitive function (S1 Table).

Forty nine of the 70 participants in the intervention group, completed the exercise program, defined as 75% or more, of the 20 scheduled exercise sessions. Median number of sessions was 20, and median number of exercises per visits was five. Among participants, 23.9% did not complete all five exercises at all visits. Walking was the most used exercise, closely followed by sit-to-stand, stepping in a grid pattern, stepping up on a box, and lunge. During the intervention period exercise levels (1–5) increased in challenge by on average +0.8 for walking (from 1.8 to 2.6), by on average +1.0 for stepping in a grid pattern (from 1.7 to 2.8), by on average +1.0 for stepping up on a box (from 1.5 to 2.5), by on average +0.5 for lunge (from 1.7 to 2.2), and by on average +0.9 for sit-to-stand (from 2.0 to 2.9).

Of the 21/70 who did not complete the exercise intervention, 12 withdrew before the first home visit, while nine participants discontinued after a median of 9 visits (minimum 3 and maximum 11 visits) due to decline in health status (n = 6), pain (n = 2), or death (n = 1).

### Baseline data

Mean age of randomized participants was 83.4 years (SD 6.1), 69.2% were women, and 72% lived alone before the fracture (Table 1).

**Table 1:** Demographics and baseline clinical characteristics (n = 143).

			Intervention n = 70		Control n = 73
Age, mean (SD)			84.0 (6.6)		82.7 (5.7)
Female sex n (%)			54 (77%)		56 (77%)
Living alone n (%)			56 (82%)		47 (65%)
Fracture (Surgery)					
	Intracapsular n (%) (arthroplasty n)		41 (59%) (33/41)		41 (56%) (34/41)
	Extracapsular n (%)		29 (41%)		32 (44%)
Use of mobility aid or assistance for walking					
	Baseline indoor n (%)		47 (67%)		45(62%)
	Baseline outdoor n (%)		66 (94%)		66 (90%)
Baseline Clinical Characteristics:		n	Median (IQR)	n	Median (IQR)
Performance-based and self-reported scales					
Mini-Mental State Examination (0–30)		69	26 (6)	72	26 (7)
Clinical Dementia Rate (sum of boxes, 0–18)		67	0 (3)	73	0 (4)
Geriatric Depression scale, (Short Form, 0–15)		68	3 (4)	68	3 (4)
Short Physical Performance Battery (SPPB, 0–12)		70	4 (3)	73	5 (5)
Barthel Index (0–20)		64	18 (3)	71	19 (4)
Nottingham E-ADL (0–66)		70	37 (28.5)	73	38 (31)
EQ-5D-3L-Index		68	0.73 (0.23)	73	0.73 (0.33)
Short FES-I (7–28)		65	10 (5)	73	10 (6)
Chalder Fatigue Scale (0–33)		61	15 (6)	62	14.5 (5)
Activity monitoring*		n	Median (IQR)	n	Median (IQR)
Upright time (min/day)		59	249.05 (208.6)	63	207.47 (178.72)
Upright events (numbers/day)		59	42.00 (20.96)	63	45.75 (17.66)
Gait		n	Median (IQR)	n	Median (IQR)
Gait speed, preferred (m/sec)		69	0.53 (0.25)	73	0.57 (0.27)
Step length (cm)**		66	40.21 (10.73)	73	42.55 (10.79)
Cadence (steps/min)**		66	86.22 (19.43)	73	89.07 (17.55)
Walk ratio (step length/cadence)		66	0.48 (0.13)	73	0.49(0.12)
Double support time (sec)		66	0.54(0.21)	73	0.49 (0.21)
Asymmetry (%)		66	10.48 (12.68)	73	9.94 (10.74)
Variability (SD) Step length (cm)		66	2.68 (1.53)	73	2.46 (1.33)
Variability (SD) Base of support (cm)		66	1.87 (1.20)	73	1.71 (1.25)

*Activity monitoring outcomes are based on 24-hour recordings from on average 5 (T1), 4.5 (T2), and 4.4 (T3) continuous days.

**Data are presented as mean (SD).

### Outcomes

**Fig 2:**
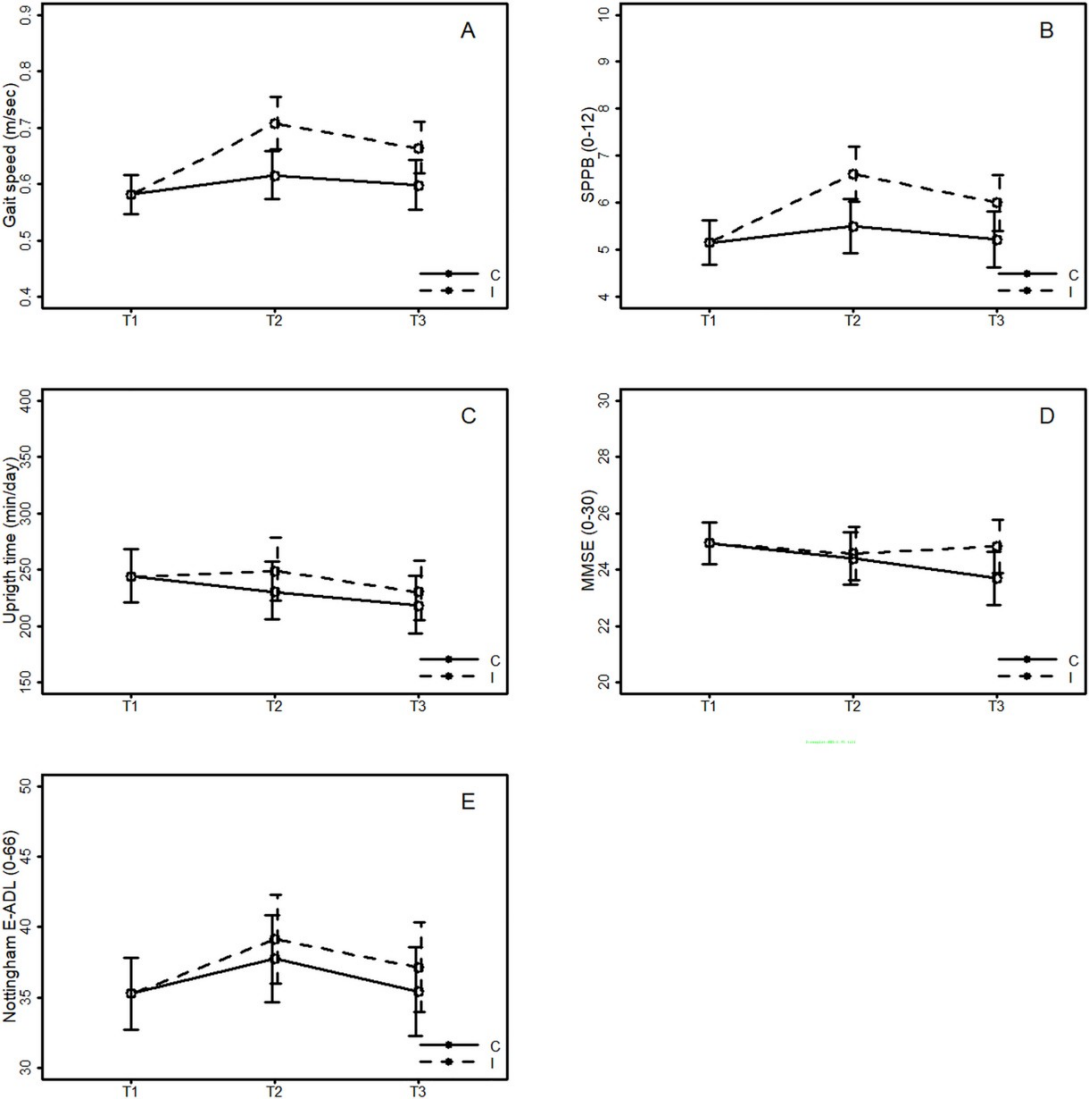
Results from the linear mixed model for the primary outcome, Gait Speed, estimated differences in mean gait speed between the interventions (Intervention (I)–Controls (C)), with 95% CI, and for four secondary outcomes (SPPB, Upright time, MMSE, and Nottingham E-ADL).

The intervention group improved relative to the control group for the primary outcome measure gait speed, from T1 to T2 (0.09 m/sec, 95% CI: 0.04 to 0.14, p<0.001) and from T1 to T3 (0.07 m/sec, 95% CI: 0.02 to 0.12, p<0.009) (Table 2 and Fig 2).

**Table 2:** Estimated between group differences in change for primary and secondary clinical outcomes and costs.

	Difference between intervention and control					
Clinical outcomes	Change from T1 to T2 *			Change from T1 to T3 *		
	Mean	95% CI	p-value	Mean	95% CI	p-value
Gait speed, preferred (m/sec)	0.09	(0.04,0.14)	<0.001	0.07	(0.02,0.12)	0.009
Gait characteristics						
Cadence (steps/min)	2.72	(-1.08,6.53)	0.161	1.13	(-2.79,5.05)	0.573
Step length (cm)	3.85	(1.57,6.14)	0.001	3.71	(1.36,6.07)	0.002
Walk ratio (step length/cadence)	0.02	(0.00,0.05)	0.099	0.02	(-0.01,0.06)	0.107
Double support time (sec)	-0.03	(-0.07,0.00)	0.087	-0.03	(-0.07,0.02)	0.205
Asymmetry (%)	-1.42	(-3.78,0.98)	0.245	-1.41	(-3.71,0.91)	0.229
Variability (SD) Step length (cm)	-0.17	(-0.56,0.21)	0.378	-0.12	(-0.57,0.32)	0.591
Variability (SD) Base of support (cm)	0.01	(-0.24,0.26)	0.947	-0.11	(-0.38,0.16)	0.426
Physical function and physical activity						
Short Physical Performance Battery (SPPB, 0–12)**	1.4	(0.8,2.1)	<0.001	1.0	(0.2,1.8)	0.017
Upright time (min/day)	18.48	(-6.61,44.40)	0.152	12.07	(-12.47,37.54)	0.346
Upright events (number/day)	4.95	(0.21,9.76)	0.039	2.29	(-2.32,7.01)	0.337
Cognitive function						
Mini Mental State Examination (0–30)**	0.2	(-0.9,1.3)	0.632	1.3	(0.3,2.5)	0.095
Geriatric Depression scale, (Short Form, 0–15)**	0.4	(-0.4,1.1)	0.368	0.0	(-0.7,0.7)	0.565
ADL-function						
Barthel Index (0–20)**	-0.1	(-0.6,0.5)	0.815	0.2	(-0.4,0.9)	0.913
Nottingham E-ADL (0–66)**	1.2	(-1.7,4.1)	0.399	1.2	(-2.8,4.9)	0.277
Other						
Geriatric Depression scale, (Short Form, 0–15)**	0.7	(-0.1,1.5)	0.184	0.1	(-0.8,1)	0.641
EQ-5D-3L-Index **	-0.01	(-0.09,0.08)	0.514	0.0	(-0.1,0.11)	0.965
Short FES-I (0–7)**	-0.2	(-1.3,0.9)	0.446	0.1	(-1.3,1.3)	0.952
Chalder Fatigue Scale (0–33)**	-1.0	(-2.2,0.2)	0.044	-0.9	(-2.5,0.4)	0.340
**Costs** ***	**Period T1 to T2**			**Period T1 to T3**		
	Mean	95% CI****	**p-value**	Mean	95% CI****	**p-value**
Physiotherapy**	1643.6	(1.3,1.9)	<0.001	1766.2	(1.3,2.2)	<0.001
Primary care						
Home-based services**	546	(-0.6,1.8)	0.080	1350.3	(-1.7,4.5)	0.125
Nursing home**	-1067.9	(-4.5,1.9)	0.947	-3222.6	(-11.3,4.4)	0.768
General Practitioner**	-19.7	(-0.1,0.1)	0.782	-107.3	(-0.3,0.1)	0.420
Hospital services						
Somatic and pshyciatric inpatient and outpatient treatment**	-383.6	(-3.2,3.8)	0.473	456.4	(-3.0,5.2)	0.663
TOTAL costs**	718.5	(-3.2,5.4)	0.062	242.9	(-8.4,8.6)	0.302

* Estimated between group differences.

** P-values from non-parametric, Mann-Whitney U-tests, and means and CIs from bootstrapping, otherwise results from linear mixed models (LMMs) on original scale or backtransformed from LMMs on transformed scales. The LMMs are adjusted for age, sex, type of fracture (extra- or intracapsular), and whether or not using a rollator.

*** Costs differences are intervention minus control. 2012 EUR

**** 95% CI in 1000 EUR

For secondary measures evidence of differences in change between groups in favour of the intervention group were found for SL (3.85 cm (95% CI: 1.57 to 6.14, p = 0.001) T1-T2 and 3.71 cm (95% CI: 1.36 to 6.07, p = 0.002) T1-T3), and SPPB (1.4 points (95% CI: 0.8 to 2.1, p<0.001) T1-T2 and 1.0 points (95% CI: 0.2 to 1.8, p = 0.017) T1-T3). Significant differences from T1 to T2 were also found for number of upright events (4.95 events (95% CI: 0.21 to 9.76, p = .039)) and chronic fatigue (-1.0 score (95% CI: -2.2 to 0.2, p = 0.044)), but these are more likely to be incidental findings due to multiple testing.

No significant differences in change between the groups were found for other measures, including ADL, upright time, cognitive function, and health-related quality of life. In total 43 persons reported one or more falls during the 12 months follow-up period (T0-T3), but no evidence of group differences was found (Pearson Chi-square test, p = 0.188).

Physiotherapy costs were significantly higher for the intervention group than for the control group for the intervention period (T1-T2) (1643.6 EUR, p<0.001) (Table 2), and from T1 to T3 (1766.2 EUR, p<0.001). From T1 to T3 no significant group difference was found for total health care costs (see S5, S6 and S7 Tables for details).

**Fig 3:**
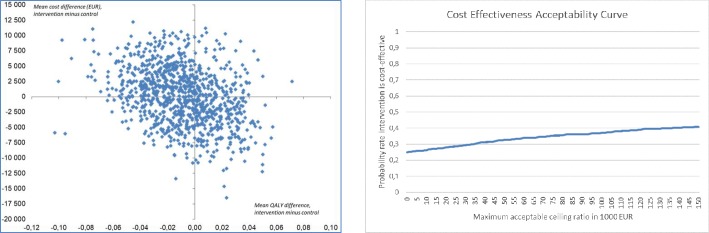
The cost-effectiveness plane (left, Fig 3A) and cost-effectiveness acceptability curve (right, Fig 3B).

The bootstrap-based QALY and cost difference estimates are shown in the cost-effectiveness plane (Fig 3A). The mean QALY difference was -0.009 (95% CI: -0.061 to 0.038). Of the 1000 replicates, 63% gave a negative QALY difference (points to the left of the vertical line, a gain in favor of control). The mean cost difference was 65.7 EUR (95% CI: -8740.4 to 9076.8), and 51% of the replicates gave higher costs for the intervention group (points above the horizontal line). The probability that the intervention was cost-effective was below 39% for any ICER ceiling ratio below 150 000 EUR per QALY gained (Fig 3B).

### Harms

Six adverse events were reported during the intervention period, of which two were serious but not related to the intervention.

## Discussion

We found both an immediate and a long-term beneficial effect on gait speed in favour of the intervention group. The effects on SL and SPPB in favour of the intervention group supported this finding. The between-group difference of 1.4 points and 1.0 points for SPPB, are both regarded as a clinically meaningful change [46]. However, we found little or no evidence of an effect on self-reported function nor upright time measured by body-worn accelerometers. Substantial meaningful changes for gait speed have been estimated to be between 0.08 to 0.14 [46], indicating that the effect in this study is of clinical importance. Larger improvements are probably required to detect self-reported mobility improvements among older people with hip-fracture [47]. This could explain why we did not find a positive effect on self-reported function or health-related quality of life. In the sample size estimate we had suggested a larger clinical effect. Our results suggest that the effect is likely to be more modest but nevertheless still within the range of clinically worthwhile [48].

Our findings suggest that there is a potential to improve gait following hip fracture by offering a homebased exercise program. Participants in the control group also received physiotherapy as part of routine practice and may partly explain the modest effect of the intervention. The effect we observed is the added effect of offering a structured 10-weeks exercise program four months after hip fracture. By offering a home-based program we probably have included vulnerable persons, who otherwise would not have received physiotherapy. Our intervention had a main focus on balance and gait exercises, as these are key aspects of mobility. Even if our program did not include progressive resistance training, the exercises in our program included functional training including muscles strength components (e.g. stepping up on a box, lunge, and sit-to-stand). Muscle strength is important for basic mobility tasks and for balance and gait control, and muscle strength was also tested as part of the SPPB that showed beneficial effects in favour of the intervention group.

We found little or no evidence of an effect on upright time or upright events. At T1 our sample had a mean gait speed of 0.6 m/s, indicating limitations in gait function in general and on outdoor mobility [49]. Former interventions with a stronger focus on daily life activities and outdoor mobility have shown effects on both mobility, ADL, and participation [50–52], and it could be argued that the potential for increasing upright time is limited as long as people do not walk outdoors.

The potential societal gains from delaying functional decline in this high-risk group is substantial. We found however that the intervention was cost neutral, indicating that higher intervention costs were outweighed by lower nursing home costs in the intervention group. Despite no differences in total health care costs and a beneficial gain in gait speed, we cannot conclude that the intervention was cost-effective from the comparison of incremental costs and QALY’s. This study cannot answer how this would have been with a longer follow-up period.

We followed participants for six months after the end of the intervention and found a lasting effect on gait speed and mobility. Among frail older adults, an accelerated decline in function is expected following an event like a hip fracture. Our finding of a lasting effect after six months could indicate that the intervention reduced the rate of decline in function. However, a longer follow-up period would be required to observe long-term effects on e.g. need for nursing home.

Our study has some limitations. We designed the study to have high external validity. However, high prevalence of frailty in this heterogenic population could be an argument for a need of more comprehensive interventions than the single-component exercise intervention in this study. Interventions beyond exercise alone is probably needed to target activity and participation specifically.

We used a two-step inclusion procedure starting with enrolment of participants from operation lists from February 2011, and inclusion of patients for the intervention four months later. However, the study was registered in June 2011 after the first randomisation of participants to the exercise intervention. Although this procedure allowed us to describe participants able or willing to participate in the exercise intervention, we acknowledge the late registration of the study as a limitation of the study design.

We planned a rather extensive test-protocol including self-reports, performance based tests, and clinical examinations. Due to burden on the participants, we reduced the test protocol for the frailest participants. Some measures that could have provided knowledge about treatment effect like measures of strength and dual task gait were only performed for the fittest participants and therefore not included in the analysis. Also, to reduce burden on the participants we used retrospective reports on falls and not fall calendars, which might explain why we did not find any effects on number of falls. Physiotherapists delivering the intervention reported adverse events directly to an independent medical team. Due to the study design a larger number of events probably have been reported in the intervention arm because of the regular contact with the physiotherapist. However, the number of serious adverse events attributable to the intervention was the same in both groups, as was the overall level of mortality. There was some evidence that those who were lost to follow-up (n = 61 at T2 and/or T3) had worse mobility at baseline than those not lost to follow-up: the mean (median) gait speed was 0.50 (0.46) and 0.60 (0.57) m/sec, respectively (p = 0.006, MWU-test). However, there was no significant difference in the proportion lost to follow-up among the control and intervention groups (p = 0.65, Chi-square test).

A major strength of this study is the clinical validity. We designed the study for high external validity. The program was performed within the frames of daily routines; the content was developed in collaboration with clinicians and lead by a group of physiotherapists representing the variety in experience and competence within the staff normally working with this patient group. The observed effect is therefore likely to be representative of routine clinical practice. In contrast to similar studies, we did also not exclude participants based on cognitive function, thus making the sample more representative for the clinical population of hip fractures. One other strength is the consecutive recruitment by operation lists and data collection prior to randomisation that allowed us to describe non-participants. Characteristics of non-participation indicated a bias towards a more fit group participating in the study compared to those who were eligible but not randomised. We also found that 30% of the participants randomised to the exercise intervention never started or completed the intervention mainly due to decline in health status, indicating a need for alternative approaches to target the most vulnerable individuals [7].

## Conclusion

We found that a relatively short home-based, supervised exercise program targeting balance and gait had an immediate and lasting small effect on gait speed and an effect on lower limb function without an increase in total health care costs. However, a tendency to include the fitter participants, a relatively high number of participants who were unable to complete the intervention and no apparent effect on daily life activities or self-reported health outcomes suggest that more comprehensive approaches are required to maximise recovery following hip-fracture.

## Supporting information

S1 Table Randomised vs. non-randomised participants (n = 223). *p-values from Mann-Whitney U-tests except for gait speed, uptime and events for which two-sample t-tests on square-root transformed data has been used.
(PDF)

S2 Table Results for estimated mean change. *) P-values from non-parametric, Mann-Whitney U-tests, and means and CIs from bootstrapping, otherwise results from linear mixed models (LMMs) on original scale or backtransformed from LMMs on transformed scales.
(PDF)

S3 Table Descriptive statistics for clinical and gait outcome variables. The data are presented as median (Inter Quartile Range, IQR) or *) mean (SD).
(PDF)

S4 Table Numbers reporting no use of walking aids/assistance during walking. (PDF)

S5 Table Unit costs in 2012 EUR. *Costs calculated from fee-for-service information from Helfo.
(PDF)

S6 Table Use of services. (PDF)

S7 Table Health and care costs per patient. **(2012 EUR, n = 143)** *) Includes physical therapist municipality and physical therapist private **) Includes occupational therapist, day based rehabilitation, ambulatory follow-up, home nursing care, home care services, safety alarm, meals on wheels, day centre ***) Include long term stay, short term stay, rehabilitation stay****) Includes hospital inpatient stay somatic ward, inpatient stay psychiatric ward, outpatient visit somatic ward and outpatient visit psychiatric ward.
(PDF)

S1 File S8 EvaHip project description Norwegian. (PDF)

S2 File S9 EvaHip project description English. (PDF)

S3 File S10 Analysis plan. (PDF)

S4 File S11 physiotherapy research international: Protocol paper. (PDF)

S5 File Checklist. (DOC)
