# Supplementary material for: Short and long-term clinical effectiveness and cost-effectiveness of a late-phase community-based balance and gait exercise program following hip fracture. The EVA-Hip Randomised Controlled Trial
Source: PLoS One. 2019 Nov 18;14(11):e0224971. doi: 10.1371/journal.pone.0224971 (PMC6860934; doi:10.1371/journal.pone.0224971)
Supplement: S1 Table — *p-values from Mann-Whitney U-tests except for gait speed, uptime and events for which two-sample t-tests on square-root transformed data has been used. (PDF) [file pone.0224971.s001.pdf]

S1 Table. Randomised vs. non-randomised participants (n=223).

	Randomized		Not randomized		Differences between groups
	n	Median (IQR)	n	Median (IQR)	p-value*
Pre-fracture data:					
Clinical Dementia Rate (sum of boxes, 0-18)	140	0 (1)	78	0.5 (4)	0.001
Barthel Index (0-20)	141	20 (2)	80	19 (3)	0.251
Nottingham E-ADL (0-66)	139	50 (25.5)	80	38.5 (30)	0.001
T1 data:					
Clinical Dementia Rate (sum of boxes, 0-18)	140	0 (3)	35	2 (6.5)	0.059
Barthel Index (0-20)	135	18 (3.5)	36	15 (10.2)	0.002
Nottingham E-ADL (0-66)	143	37 (31)	37	19 (37)	0.007
Mini Mental State Examination (0-30)	141	26 (7)	24	25 (7.2)	0.482
Short Physical Performance Battery (SPPB, 0-12)	143	4 (4)	30	3 (5)	0.016
Gait speed, preferred (m/sec)	142	57.8 (36.1)	25	46.3 (52.7)	0.705
Upright time (min/day)	122	218.5 (198)	23	182.1 (200.5)	0.054
Upright Events (number/day)	122	44 (20.2)	23	43.3 (37.6)	0.085

*p-values from Mann-Whitney U-tests except for gait speed, uptime and events for which two-sample t-tests on square-root transformed data has been used.
