# Supplementary material for: Short and long-term clinical effectiveness and cost-effectiveness of a late-phase community-based balance and gait exercise program following hip fracture. The EVA-Hip Randomised Controlled Trial
Source: PLoS One. 2019 Nov 18;14(11):e0224971. doi: 10.1371/journal.pone.0224971 (PMC6860934; doi:10.1371/journal.pone.0224971)
Supplement: S2 Table — *) P-values from non-parametric, Mann-Whitney U-tests, and means and CIs from bootstrapping, otherwise results from linear mixed models (LMMs) on original scale or backtransformed from LMMs on transformed scales. (PDF) [file pone.0224971.s002.pdf]

S2 Table. Results for estimated mean change. *) P-values from non-parametric, Mann-Whitney U-tests, and means and CIs from bootstrapping, otherwise results from linear mixed models (LMMs) on original scale or backtransformed from LMMs on transformed scales.

	Change from T1 to T2						Change from T1 to T3					
	Intervention			Control			Intervention			Control		
	Mean	95% CI	p-value	Mean	95% CI	p-value	Mean	95% CI	p-value	Mean	95% CI	p-value
Gait speed, preferred (m/sec)	0.13	(0.09,0.16)	<0.001	0.03	(0.00,0.07)	0.051	0.08	(0.05,0.12)	<0.001	0.02	(-0.02,0.05)	0.349
Upright time (min/day)	4.82	(-13.91,24.61)	0.614	-13.66	(-31.35,3.73)	0.132	-13.78	(-31.8,4.58)	0.144	-25.85	(-43.85,-7.61)	0.006
Events (no./day)	4.60	(1.10,8.20)	0.010	-0.34	(-3.64,2.96)	0.838	-0.05	(-3.44,3.38)	0.975	-2.34	(-5.67,1.04)	0.179
Step Length (cm)	4.84	(3.18,6.5)	<0.001	0.99	(-0.65,2.62)	0.236	4.71	(3.02,6.41)	<0.001	1.00	(-0.69,2.70)	0.247
Cadence (steps/min)	7.14	(4.36,9.93)	<0.001	4.42	(1.68,7.16)	0.002	4.96	(2.11,7.81)	0.001	3.83	(0.99,6.68)	0.008
Asymmetry (%)	-1.96	(-3.73,-0.12)	0.036	-0.54	(-2.39,1.38)	0.571	-3.20	(-4.92,-1.41)	<0.001	-1.79	(-3.60,0.07)	0.062
SPPB (0-12) *)	1.6	(1.1,2.2)	<0.001	0.2	(-0.2,0.5)	0.397	1	(0.4,1.6)	0.003	-0.1	(-0.6,0.5)	0.621
MMSE (0-30) *)	-0.4	(-1.2,0.4)	0.302	-0.6	(-1.3,0.1)	0.140	-0.1	(-0.8,0.7)	0.697	-1.4	(-2.4,-0.6)	0.006
CDR (sum of boxes, 0-18) *)	0.2	(-0.3,0.8)	0.374	-0.2	(-0.7,0.3)	0.661	0	(-0.5,0.5)	0.951	0	(-0.5,0.4)	0.874
Barthel Index (0-20) *)	-0.1	(-0.5,0.2)	0.959	0	(-0.6,0.4)	0.350	0	(-0.5,0.4)	0.922	-0.2	(-0.7,0.2)	0.515
Nottingham E-ADL (0-66) *)	3.8	(1.8,6.1)	0.001	2.6	(0.8,4.7)	0.014	1.3	(-2.1,4.1)	0.183	0.1	(-2.3,2.3)	0.926
GDS (Short Form, 0-15) *)	0.2	(-0.4,0.8)	0.607	-0.5	(-1.1,0.1)	0.138	0.3	(-0.2,0.9)	0.299	0.2	(-0.5,0.9)	0.801
Variability (SD) Step Length (cm)	0.14	(-0.14,0.43)	0.336	0.31	(0.03,0.62)	0.034	0.5	(0.18,0.83)	0.002	0.62	(0.29,0.98)	<0.001
Variability (SD) Base of support (cm)	0.06	(-0.12,0.25)	0.526	0.05	(-0.13,0.24)	0.580	0.06	(-0.13,0.26)	0.553	0.17	(-0.03,0.38)	0.098
EQ-5D-3L-Index *)	0.03	(-0.03,0.08)	0.059	0.04	(-0.03,0.10)	0.156	-0.03	(-0.10,0.02)	0.717	-0.03	(-0.13,0.05)	0.732
Short FES-I (0-7) *)	-0.8	(-1.7,0.1)	0.042	-0.6	(-1.2,0.1)	0.061	-0.8	(-2,0.2)	0.202	-0.9	(-1.7,-0.3)	0.023
Chalder Fatigue Scale (0-33) *)	-1	(-1.8,-0.1)	0.021	0	(-0.8,0.8)	0.706	-0.2	(-1.5,0.8)	0.803	0.7	(-0.2,1.7)	0.271
