# Supplementary material for: Short and long-term clinical effectiveness and cost-effectiveness of a late-phase community-based balance and gait exercise program following hip fracture. The EVA-Hip Randomised Controlled Trial
Source: PLoS One. 2019 Nov 18;14(11):e0224971. doi: 10.1371/journal.pone.0224971 (PMC6860934; doi:10.1371/journal.pone.0224971)
Supplement: S3 Table — The data are presented as median (Inter Quartile Range, IQR) or *) mean (SD). (PDF) [file pone.0224971.s003.pdf]

S3 Table. Descriptive statistics for clinical and gait outcome variables. The data are presented as median (Inter Quartile Range, IQR) or *) mean (SD).

	Intervention						Control					
	T1		T2		T3		T1		T2		T3	
	n	Mean (SD) / Median (IQR)	n	Mean (SD) / Median (IQR)	n	Mean (SD) / Median (IQR)	n	Mean (SD) / Median (IQR)	n	Mean (SD) / Median (IQR)	n	Mean (SD) / Median (IQR)
Gait speed, preferred (m/sec)	69	0.53 (0.25)	61	0.67 (0.3)	55	0.63 (0.27)	73	0.57 (0.27)	61	0.62 (0.28)	55	0.61 (0.32)
Uprigth time (min/day)	59	249.05 (208.6)	50	241.29 (175.74)	49	217.98 (162.73)	63	207.47 (178.72)	57	235.1 (189.14)	49	235.52 (171.80)
Events (no./day)	59	42.00 (20.96)	50	48.58 (24.09)	49	45.17 (18.75)	63	45.75 (17.66)	57	47.75 (26.33)	49	44.17 (20.25)
Step Length (cm) *)	66	40.21 (10.73)	57	45.50 (10.84)	54	45.50 (11.00)	73	42.55 (10.79)	58	44.38 (12.04)	53	44.82 (12.45)
Cadence (steps/min) *)	66	86.22 (19.43)	57	93.51 (17.64)	54	92.12 (15.98)	73	89.07 (17.55)	58	94.94 (14.71)	53	95.24 (16.27)
Asymmetry (%)	66	10.48 (12.68)	57	7.41 (9.07)	54	6.45 (9.69)	73	9.94 (10.74)	58	8.07 (11.07)	53	7.40 (10.65)
SPPB (0-12)	70	4 (3)	61	7 (5)	56	6 (5)	73	5 (5)	62	6 (4)	57	6 (6)
MMSE (0-30)	69	26 (6)	57	26 (5)	56	26.5 (7)	72	26 (7)	60	26 (5)	53	26 (6)

CDR (sum of boxes, 0-18)	67	0 (3)	58	0 (3)	52	0 (3)	73	0 (4)	58	0 (2.8)	54	0 (3)
Barthel Index (0-20)	64	18 (3)	61	18 (4)	55	18 (3)	71	19 (4)	62	19 (3)	57	19 (4)
Nottingham E-ADL (0-66)	70	37 (28.5)	60	47 (27.8)	55	41 (26.5)	73	38 (31)	62	40.5 (18.8)	57	40 (28)
GDS (short Form, 0-15)	68	3 (4)	58	3 (3.8)	52	2 (4.2)	68	3 (4)	56	2 (4.2)	53	2 (5)
Variability (SD) Step Length (cm)	66	2.68 (1.53)	57	2.72 (1.06)	54	3.10 (1.50)	73	2.46 (1.33)	58	3.02 (1.37)	53	3.20 (1.64)
Variability (SD) Base of support (cm)	66	1.87 (1.20)	57	1.87 (1.25)	54	1.92 (1.18)	73	1.71 (1.25)	58	1.88 (0.79)	53	1.84 (0.90)
EQ-5D-3L-Index	68	0.73 (0.23)	60	0.73 (0.23)	57	0.73 (0.23)	73	0.73 (0.33)	60	0.73 (0.16)	61	0.73 (0.23)
Short FES-I (0-7)	65	10 (5)	59	9 (5)	51	9 (3.5)	73	10 (6)	60	8.5 (3)	54	9 (3.8)
Chalder Fatigue Scale (0-33)	61	15 (6)	55	15 (5.5)	47	15 (6.5)	62	14.5 (5)	54	15 (6.5)	48	15 (6)
