# Supplementary material for: Short and long-term clinical effectiveness and cost-effectiveness of a late-phase community-based balance and gait exercise program following hip fracture. The EVA-Hip Randomised Controlled Trial
Source: PLoS One. 2019 Nov 18;14(11):e0224971. doi: 10.1371/journal.pone.0224971 (PMC6860934; doi:10.1371/journal.pone.0224971)
Supplement: S4 Table — (PDF) [file pone.0224971.s004.pdf]

S4 Table. Numbers reporting no use of walking aids/assistance during walking.

	Intervention (n=70)			Controls (n=73)		
	n	n (%)	*missing*	n	n (%)	*missing*
Pre-fracture indoor	68	50 (73.5)	2	71	56 (78.9)	2
Pre-fracture outdoor	67	31 (46.3)	3	69	45 (65.2)	4
Baseline indoor	70	23 (32.9)	0	73	28 (38.4)	0
Baseline outdoor	70	4 (5.7)	0	73	7 (20.5)	0
Post-test indoor	62	32 (51.6)	8	63	37 (58.7)	10
Post-test outdoor	62	11 (17.7)	8	62	13 (21.0)	11
Follow-up indoor	55	25 (45.5)	15	57	35 (61.4)	16
Follow-up outdoor	55	7 (12.7)	15	57	12 (21.1)	16
