# Supplementary material for: Short and long-term clinical effectiveness and cost-effectiveness of a late-phase community-based balance and gait exercise program following hip fracture. The EVA-Hip Randomised Controlled Trial
Source: PLoS One. 2019 Nov 18;14(11):e0224971. doi: 10.1371/journal.pone.0224971 (PMC6860934; doi:10.1371/journal.pone.0224971)
Supplement: S5 Table — *Costs calculated from fee-for-service information from Helfo. (PDF) [file pone.0224971.s005.pdf]

S5 Table. Unit costs in 2012 EUR.

Services type	Type of unit	Unit cost (EUR)	Source of information
**Physiotherapy**			
Physical therapist - municipality	Hours	101	Municipality of Trondheim
Physical therapist - private	Hours	*	The Norwegian Health Economics Administration (Helfo)
**Home based services**			
Occupational therapist	Hours	96	Municipality of Trondheim
Long term stay	Days	302	Municipality of Trondheim
Ambulatory follow-up	Hours	94	Municipality of Trondheim
Home nursing care	Hours	97	Municipality of Trondheim
Home care services	Hours	95	Municipality of Trondheim
Safety alarm	Months	5	Municipality of Trondheim
Meals on wheels	Months	21	Municipality of Trondheim
Visits to daycentre	Months	666	Municipality of Trondheim
**Nursing home**			
Short term stay - rehabiliation	Days	385	Municipality of Trondheim
Short term stay	Days	321	Municipality of Trondheim
Day based rehabilitation	Days	196	Municipality of Trondheim
**General practitioner**	Visits	*	The Norwegian Health Economics Administration (Helfo)
**Hospital services**			
Inpatient stay - somatic ward	Days	1291	St Olavs hospital, from local cost-per-patient system
Inpatient stay - psychiatric ward	Days	1168	Norwegian Directorate of Health, average cost per diem St Olavs hospital.
Outpatient visit - somatic ward	Visits	245	St Olavs hospital, from local cost-per-patient system
Outpatient visit - psychiatric ward	Visits	343	Norwegian Directorate of Health, average cost per diem St Olavs hospital.

**Costs calculated from fee-for-service information from Helfo.*
