# Supplementary material for: Short and long-term clinical effectiveness and cost-effectiveness of a late-phase community-based balance and gait exercise program following hip fracture. The EVA-Hip Randomised Controlled Trial
Source: PLoS One. 2019 Nov 18;14(11):e0224971. doi: 10.1371/journal.pone.0224971 (PMC6860934; doi:10.1371/journal.pone.0224971)
Supplement: S6 Table — (PDF) [file pone.0224971.s006.pdf]

## S6 Table. Use of services.

| | | Intervention | | Control | |
|---|---|---|---|---|---|
| **Services type** | **Type of unit** | **Mean #** | **Sd** | **Mean #** | **Sd** |
| **Physiotherapy** | | | | | |
| Physical therapist – municipality | Hours | 24,4 | 13,7 | 4,3 | 8,0 |
| Physical therapist - private | Visits | 3,9 | 8,8 | 6,4 | 12,3 |
| **Home based services** | | | | | |
| Occupational therapist | Hours | 0,5 | 1,6 | 0,2 | 0,9 |
| Day based rehabilitation | Days | 0,5 | 4,4 | 1,8 | 15,0 |
| Ambulatory follow-up | Hours | 0,1 | 0,6 | 0,1 | 0,5 |
| Home nursing care | Hours | 18,4 | 45,0 | 14,2 | 42,5 |
| Home care services | Hours | 48,1 | 77,0 | 41,3 | 71,2 |
| Safety alarm | Months | 7,6 | 1,6 | 6,7 | 2,8 |
| Meals on wheels | Months | 0,8 | 2,3 | 0,2 | 1,3 |
| Daycare centre | Months | 1,5 | 3,0 | 1,1 | 2,7 |
| **Nursing home** | | | | | |
| Long term stay | Days | 24,5 | 67,6 | 33,4 | 74,9 |
| Short term stay | Days | 6,1 | 12,7 | 6,7 | 20,4 |
| Rehabilitation stay | Days | 2,0 | -9,6 | 2,0 | 9,6 |
| **General practitioner** | Visits | 11,4 | 10,6 | 12,5 | 10,5 |
| **Hospital services** | | | | | |
| Inpatient stay - somatic ward | Days | 2,6 | 5,8 | 3,0 | 5,8 |
| Inpatient stay - psychiatric ward | Days | 0,9 | 7,9 | - | 7,9 |
| Outpatient visit - somatic ward | Visits | 1,6 | 2,7 | 2,6 | 2,7 |
| Outpatient visit - psychiatric ward | Visits | 0,4 | 2,5 | 0,1 | 2,5 |
