# Supplementary material for: Short and long-term clinical effectiveness and cost-effectiveness of a late-phase community-based balance and gait exercise program following hip fracture. The EVA-Hip Randomised Controlled Trial
Source: PLoS One. 2019 Nov 18;14(11):e0224971. doi: 10.1371/journal.pone.0224971 (PMC6860934; doi:10.1371/journal.pone.0224971)
Supplement: S7 Table — (2012 EUR, n = 143) *) Includes physical therapist municipality and physical therapist private **) Includes occupational therapist, day based rehabilitation, ambulatory follow-up, home nursing care, home care services, safety alarm, meals on wheels, day centre ***) Include long term stay, short term stay, rehabilitation stay****) Includes hospital inpatient stay somatic ward, inpatient stay psychiatric ward, outpatient visit somatic ward and outpatient visit psychiatric ward. (PDF) [file pone.0224971.s007.pdf]

S7 Table. Health and care costs per patient. (2012 EUR, n=143).

	Period T1 to T2				Period T1 to T3			
	Intervention		Control		Intervention		Control	
	Mean	SD	Mean	SD	Mean	*SD*	Mean	*SD*
**Physiotherapy*)**	2239	1231	596	728	2721	1404	955	1206
Primary care								
**Home based services**)**	2813	3758	2267	3379	7454	9788	6103	9187
**Nursing home***)**	4010	8754	5078	10387	10501	20817	13724	25451
**General Practitioner**	227	231	247	284	550	469	658	675
Hospital services								
**Sum inpatient and outpatient****)**	2116	11431	2500	8909	4993	13331	4537	10290
**TOTAL costs**	11406	13765	10687	13417	26219	25468	25976	28631

*) Includes physical therapist municipality and physical therapist private
**) Includes occupational therapist, day based rehabilitation, ambulatory follow-up, home nursing care, home care services, safety alarm, meals on wheels, day centre
***) Include long term stay, short term stay, rehabilitation stay
****) Includes hospital inpatient stay somatic ward, inpatient stay psychiatric ward, outpatient visit somatic ward and outpatient visit psychiatric ward
