# Supplementary material for: Short and long-term clinical effectiveness and cost-effectiveness of a late-phase community-based balance and gait exercise program following hip fracture. The EVA-Hip Randomised Controlled Trial
Source: PLoS One. 2019 Nov 18;14(11):e0224971. doi: 10.1371/journal.pone.0224971 (PMC6860934; doi:10.1371/journal.pone.0224971)
Supplement: S1 File — (PDF) [file pone.0224971.s008.pdf]

Prosjektbeskrivelse EVA-HIP-studien

Evaluation of rehabilitation after hip fracture

Effekt av rehabiliteringstiltak på gangfunksjon og aktivitetsnivå etter hoftebrudd

1. Innledning

Norge har verdens høyeste forekomst av hoftebrudd med i underkant av 10 000 tilfeller hvert år. Et hoftebrudd er estimert til å koste mellom 250 000 og 1,1 mill kr det første året (1). Et mer effektivt rehabiliteringstilbud vil trolig kunne gi stor samfunnsøkonomisk gevinst. Hoftebrudd rammer i all hovedsak eldre personer, og hoftebruddspasientene har vanligvis et sammensatt sykdomsbilde. Et hoftebrudd er forbundet med en betydelig reduksjon i funksjons- og aktivitetsnivå, økt dødelighet og økt hjelpebehov. I løpet av det første året etter bruddet vil 30 % av pasientene være døde (2;3), og 20 % av pasientene som var hjemmeboende før bruddet vil bo i sykehjem. Mer enn halvparten vil ha opplevd nye fall, og 30 % har pådratt seg skader som følge av nye fall (4).

En observasjonsstudie gjennomført av Forskningsgruppe for geriatri (FFG) i 2007/2008 viser at flertallet av eldre pasienter med hoftebrudd får betydelig økt hjelpebehov og redusert funksjonsnivå etter bruddet (5). Dette gjelder også pasienter med relativt høyt funksjonsnivå før bruddet. Dette er i samsvar med annen dokumentasjon og viser at et hoftebrudd er en dramatisk hendelse med store konsekvenser for den som rammes. Det er også godt dokumentert at gangfunksjon blir betydelig svekket etter et hoftebrudd. Dette er høyst sannsynlig en viktig årsak til økt hjelpebehov og økt falltendens etter bruddet. Samtidig viser flere studier at mobilitet og gangfunksjon kan bedres som følge av målrettet intervensjon (3), noe som indikerer et potensial for å oppnå bedre gangfunksjon enn hva tilfellet er i dag.

Fysisk aktivitet er en trolig også viktig for gjenvinning av fysisk funksjon etter et hoftebrudd. Likevel finnes svært lite kunnskap om hoftebruddspasientenes fysisk aktivitet i løpet av rehabiliteringsperioden og hvor stor dose og intensitet av fysisk aktivitet som er nødvendig for å gjenvinne tidligere funksjon. Siden de fleste ikke gjenvinner gangfunksjon, kan man anta at totalmengden fysisk aktivitet er for liten. Små kroppsbårne aktivitetssensorer kan i dag benyttes for å måle fysisk aktivitet i hverdagslivet over flere dager. Denne type informasjon

vil være nyttig for å få mer kunnskap om faktorer som kan bedre effekten av rehabilitering etter hoftebrudd.

Erfaringer og tilbakemelding fra pasienter som har deltatt i observasjonsstudien nevnt foran og pasienter som deltar i en pågående klinisk kontrollert randomisert studie (Hip-Unit-studien) ved St.Olavs Hospital og NTNU, kan tyde på at rehabiliteringstilbudet og oppfølgingen etter bruddet er fragmentert og lite tilpasset den enkelte.

For å sikre en god og helhetlig oppfølging gjennom pasientforløpet, fra innleggelse i sykehuset til rehabiliteringen blir avsluttet, er det behov for å fokusere på samhandling mellom de ulike tjenestene og forvaltningsnivåene. Tiltakskjeden inkluderer overgangsfaser der pasienten flyttes fra sykehus, til opptreningsopphold eller direkte til hjemmet. Målsettingen med denne studien er å sikre at eldre pasienter med hoftebrudd får helhetlig og individuelt tilpasset oppfølging gjennom rehabiliteringforløpet, for å sikre best mulig gjenvinning av gangfunksjon. For å få til dette vil studien ha særskilt fokus på tiltak basert på individuelle behov og med særlig vekt på fysisk aktivitet.

2. Faggruppe/miljø

Forskningsgruppe for geriatri (FFG) er en tverrfaglig gruppe bestående av 13 personer, hvorav tre er seniorforskere; Olav Sletvold, Jorunn Lægdheim Helbostad og Ingvild Saltvedt og 11 har mastergradsutdanning eller høyere. FFG har særlig erfaring med klinisk kontrollerte og randomiserte forsøk. Første større studie sammenlignet nytteverdien av å behandle skrøpelige geriatriske pasienter i en spesialavdeling for geriatri med en indremedisinsk avdeling ved St. Olavs Hospital, se Ingvild Saltvedts doktoravhandling (NTNU 2002) (6). Gjennomføringen av dette prosjektet ble støttet av Sosial- og Helsedepartementet og Norges forskningsråd. Neste studie sammenlignet ulike typer trening av hjemmeboende og skrøpelige eldre i Trondheim, se Jorunn L. Helbostads doktoravhandling (UiB 2004) (7).

Fra 2005 har FGG særlig fokusert på eldre med gangvansker, falltendens og fallskader, særlig hoftebruddpasienter. Prosjektet "Assessment and treatment of older people at risk of falling" (2006-2008) og som blant annet inneholdt en observasjonsstudie av hoftebruddpasienter i 3 måneder etter operasjon, ble støttet med NOK 2 millioner av Norges forskningsråd. Samme prosjekt evaluerte dessuten nytteverdien av kataraktoperasjoner med henblikk på

gangfunksjon og falltendens. I 2008 fikk FFG en NFR-bevilgning på NOK 3 millioner for å gjennomføre et prosjekt med sammenligning av tradisjonell behandling av hoftebruddpasienter i en ortopedisk avdeling mot en ortogeriatrisk avdeling. Mulighetene for å gjennomføre EVA-HIP-prosjektet etter plan er blitt ytterligere styrket i løpet av 2010 etter Samarbeidsorganets bevilgning av infrastrukturmidler for Ganglab på NOK 7,5 millioner over en 5 års-periode og at INM, DMF, NTNU har definert "Bevegelsesrelaterte tilstander" som prioritert satsingsområde ved instituttet.

Eva-Hip-studien gjennomføres i tett samarbeid med helsetjenesten i Trondheim kommune ved kommuneoverlege Helge Garåsen. Enhet for fysioterapi, Trondheim kommune ved leder Anne E. Hansen og fagkoordinator for eldre, Sylvi Sand, og Randi Granbo (HIST, fagkoordinator i Trondheim kommune) vil være viktige samarbeidspartnere i forhold til utvikling av tjenestetilbudet i en slik rehabiliteringskjede og implementering av intervensjonen i kommunen.

3. Problemstilling / Målsetting og forskningsspørsmål:

Det overordnede formålet med Eva-Hip-studien er å forbedre rehabiliteringstilbudet til eldre etter hoftebrudd etter utskrivelse fra sykehuset. Prosjektet består av to delstudier. Del 1 omfatter forløpet fra utskriving og fram til fire måneder etter bruddet, og del 2 er en fysisk-treningsstudie som starter fire måneder etter bruddet. I det følgende omtales de to delstudiene henholdsvis som forløpsstudien og treningsstudien.

Forløpsstudien har til hensikt å se om et utvidet helhetlig pasientforløp kan gi en effekt på aktivitetsnivå og gangfunksjon gjennom å introdusere faste tidspunkt for funksjonsvurderinger etter utskrivelse fra sykehuset og øke kunnskap om og ha fokus på fysisk aktivitet og gjenvinning av gangfunksjon. Treningsstudien har til hensikt å øke gangfunksjons- og aktivitetsnivå etter hoftebrudd gjennom et spesifikt treningstilbud 4-6 måneder etter bruddet. Målsettingen er å undersøke hvilken effekt spesifikk trening har på aktivitetsnivå og gangfunksjon etter hoftebrudd.

Forskningsspørsmål:

1. Hva er effekten av et nytt og helhetlig pasientforløp som fokuserer på rehabilitering sammenlignet med standard forløp etter utskrivelse av hoftebruddpasienter fra sykehus på mengde og type fysisk aktivitet?
2. Hva er effekten av et nytt og helhetlig pasientforløp som fokuserer på rehabilitering sammenlignet med standard forløp etter utskrivelse av hoftebruddpasienter fra sykehus på gangfunksjon?
3. Hva er effekten av et individuelt tilpasset styrke- og balanse-treningsprogram gjennomført 4-6 måneder etter hoftebrudd sammenlignet med standard forløp på fysisk aktivitet og gangfunksjon?

4. Metode

Målgruppe

Målgruppen for denne studien er eldre hoftebruddspasienter ≥ 70 år som var hjemmeboende og hadde gangfunksjon (10 meter med eller uten ganghjelpemiddel) før bruddet. Personer bosatt i Trondheim kommune og innlagt St. Olavs Hospital med hoftebrudd i øvre femurende (mediale, per- og subtrochantære), inkluderes. Pasienter med kort forventet levetid (< 3 mnd), multitraumer eller patologiske brudd blir ekskludert. Kognitiv svikt er ikke et eksklusjonskriterium, da eldre med kognitiv svikt er en gruppe som er spesielt interessante i forhold til å evaluere rehabiliteringsinnsats. For treningsstudien ekskluderes pasienter som ikke kan gå 10 meter 4 måneder etter bruddet eller som etter en medisinsk undersøkelse er vurdert til at trening er kontraindisert.

Design

Prosjektet består av to delstudier; forløpsstudien og treningsstudien. Forløpsstudien fokuserer på pasientforløpet de første fire måneder etter bruddet og evaluerer et utvidet helhetlig pasientforløp med faste tidspunkt for funksjonsvurderinger etter utskrivelse fra sykehuset. Denne studien gjennomføres som en kvasi-eksperimentell kohorte-studie med historiske kontroller fra den pågående Hip-Unit-studien. Ett hundre og tretti nye pasienter inkluderes i kasusgruppen.

Ved endt oppfølging fire måneder etter bruddet introduseres treningsstudien, hvor deltakerne fra forløpsstudien randomiseres til fysisk trening eller konvensjonell oppfølging. Dette er

tenkt som en klinisk randomisert studie, med parallelt gruppedesign, der trening sammenlignes med ordinær oppfølging. Det er planlagt stratifisert blokkrandomisering på operasjonsmetode og bruk av ganghjelpemiddel inne før bruddet (Ja/Nei). Testere i prosjektet vil være blindet for deltakernes gruppetilhørighet. Randomisering skjer gjennom et web-basert program i regi av Enhet for anvendt klinisk forskning ved NTNU.

Prosedyrer

Figur 1 viser flyt-diagram over Eva-Hip-studien, både forløpsstudien og treningsstudien. Pasient/operasjonslister ved Ortopedisk avdeling og Avdeling for geriatri ved St. Olavs hospital gjennomgås for å identifisere aktuelle deltakere til studien. De aktuelle hoftebruddspasientene forespørres av sykehuspersonale om deltakelse i studien 3-7 dager etter operasjon. Prosjektregistreringer blir gjennomført ved inklusjon (under indexoppholdet), og inkluderer opplysninger fra pasientjournal og informasjon innhentet gjennom strukturert intervju med pasient/pårørende. Fire måneder etter hoftebruddet avsluttes forløpsstudien, og deltakerne gjennomfører aktivitets- og funksjonsregistreringer. Deltakere som oppfyller inklusjon til treningsgruppestudien randomiseres så til treningsgruppe eller kontrollgruppe, der treningsgruppen gjennomfører spesifikk trening 2 ganger i uka i 10 uker, mens kontrollgruppen får ordinær oppfølging. Tolv måneder etter bruddet gjennomføres aktivitets- og funksjonsregistreringer.

Prosjektregistreringene vil foregå ved Ganglaben på Institutt for nevromedisin, DMF, NTNU. Hvis dette ikke lar seg gjøre, benyttes et redusert testbatteri der pasienten oppholder seg (institusjon eller i hjemmet). Aktivitetsmålere bæres på kroppen over 6 dager i pasientens vante omgivelser og hentes inn av prosjektmedarbeider.

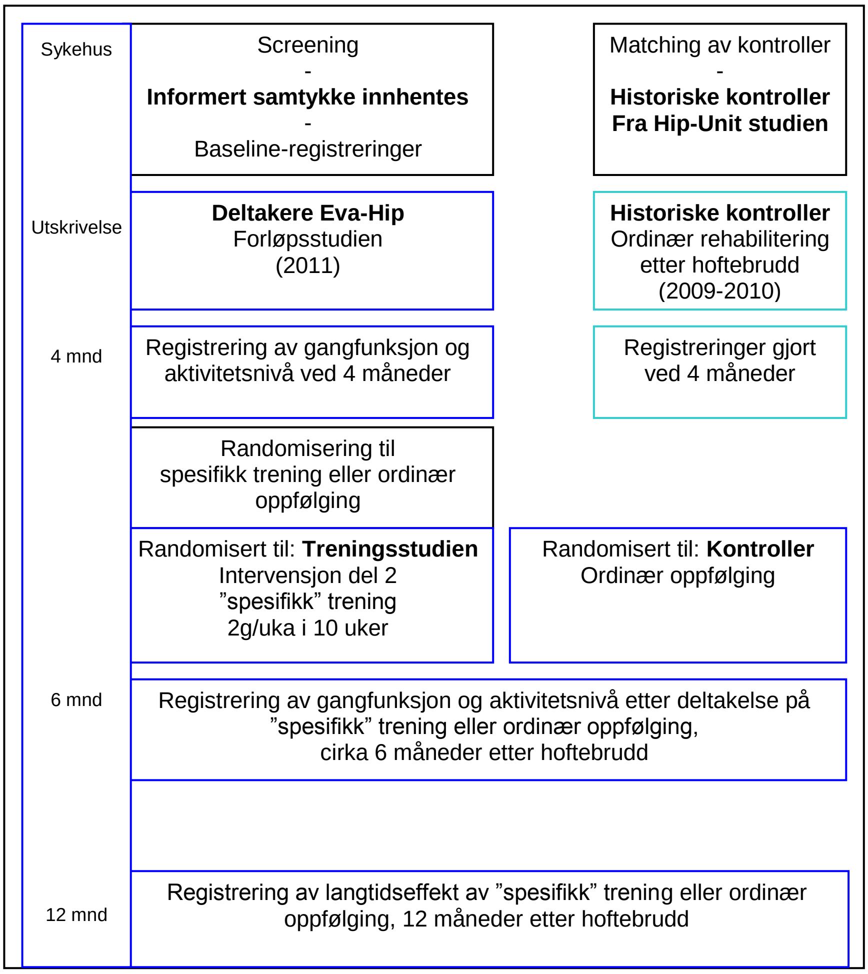

Figur 1. Flytskjema over inklusjon og intervensjon gjennom hele Eva-Hip

Intervensjon

Forløpsstudien med tidlig varsling, 3-dagersbesøk, 4-ukersvurdering

Forløpsstudien forankres i den organisasjonsstrukturen som nå etableres som del av tjenestetilbudet i Trondheim kommune gjennom ”Helhetlig pasientforløp” (HPH). HPH er pleie- og omsorgstjenestens verktøy for å sikre et optimalt pasientforløp for personer som utskrives fra sykehus til hjemmet og som skal ”breddes” i alle bydeler i kommunen innen desember 2010. Mange hoftebruddspasienter vil ikke omfattes av HPH fordi de ikke har et pleie- og omsorgstjenestetilbud. Dette gjelder for eksempel de som kun har hjemmehjelp eller trygghetsalarm, de som er på rehabiliteringsopphold før de reiser hjem eller de som ved utskrivelse reiser direkte til hjemmet og som ikke er registrert som brukere av helsetjenester. For å sikre et optimalt pasientforløp for alle hoftebruddspasientene i Eva-Hip-prosjektet, vil kommunefysioterapeutene ha et spesielt ansvar for å benytte HPH-verktøyet for funksjonsvurderinger til faste tidspunkt gjennom pasientløpet.

For at fysioterapitjenesten i kommunen skal kunne gjennomføre tidlige funksjonsvurderinger for hoftebruddspasienter etter utskrivelse fra sykehuset, etableres en ny rutine om tidlig varsling fra sykehuset. Denne rutinen innebærer at det sendes melding til henvisningsanvarlig fysioterapeut om deltakere inkludert i prosjektet. Etter at henvisningsansvarlig fysioterapeut i riktig bydel har fått varsling om at en pasient er inkludert og deltar i studien, blir denne registrert som aktiv bruker. Kommunefysioterapeuten får første oppdatering om pasienten ved å lese notat fra vurderingsmøte på sykehuset og plan for utskrivelse sjekkes samtidig. De fleste hoftebruddspasientene vil trolig innom et korttidsopphold på helsehus eller rehabiliteringsinstitusjon før hjemreise, men noen reiser direkte hjem etter sykehusoppholdet og andre får plass på sykehjem. Forventet tidspunkt for hjemreise fra rehabiliteringsinstitusjon eller sykehjem innhentes av behandlingsansvarlig fysioterapeut i kommunen, og funksjonsvurdering vil gjennomføres 3 dager etter utskrivelse til hjemmet slik at tiltak kan settes i gang tidlig. Kommunefysioterapeut gjennomfører så en 4-ukers samtale for å følge opp og vurdere videre tilbud. Deltakere i prosjektet som får plass på sykehjem får samme oppfølging med vurdering ved 3 dager og 4-uker.

For å øke fagkunnskapen om hoftebrudd hos fysioterapeuter som skal ha kontakt med hoftebruddspasienter i hele pasientforløpet, gjennomføres det i forkant av prosjektstart fagdager for fysioterapeuter med fokus på rehabilitering etter hoftebrudd. Målet er å sette fokus på samhandling og erfaringsutveksling fra fysioterapeuter som arbeider med pasientgruppen på ulike tjenestenivå. Fagdagene involverer fysioterapeuter på sykehus, rehabiliteringsintitusjoner, helsehus, institutter og kommune.

Treningsstudien

Andre del av rehabiliteringsstudien består av en fysioterapi-intervensjon som skjer i tett samarbeid med kommunens fysioterapitjeneste og fysikalske institutter i kommunen. Intervensjonen består av en kort og intensiv treningsbolk x 2 per uke over 10 uker utført av fysioterapeuter i Enhet for fysioterapi, Trondheim Kommune, eller ved et fysikalsk institutt i kommunen, 4-6 måneder etter hoftebruddet. Treningen er individuell og fokuserer på fysisk aktivitetsnivå og gangfunksjon. Det konkrete innholdet utarbeides i samarbeid med fysioterapeuter i Trondheim kommune som et tjenesteutviklingsprosjekt. Aktuelle deltakere i

utarbeidelse av treningsinnholdet rekrutteres gjennom fagdagene som blir holdt rett før prosjektstart til Eva-Hip.

Utvalgsstørrelse og styrkeberegning

Utvalgsstørrelse og styrkeberegning i denne studien baseres på data fra observasjonsstudien og Hip-Unit-studien som allerede er gjennomført. Pasientgruppen eldre med hoftebrudd har høy morbiditet og mortalitet, og frafall vil derfor være en særlig utfordring i denne studien. Andre treningsstudier på hoftebruddspasienter har rapportert opp til 30 % frafall i intervensjonsperioden (8).

Det er bred enighet i litteraturen om at ganghastighet er et av de beste tilgjengelige mål på funksjonsnivå og helse hos skrøpelige eldre (9). Materialet fra observasjonsstudien viste gjennomsnittelig ganghastighet 3 måneder etter bruddet på 0.5 m/s med standardavvik på 0.2 m/s ved selvvalgt ganghastighet. Med 90 % power og 5 % signifikans nivå, og en forskjell på 0.13 m/s i ganghastighet mellom kontroll og intervensjonsgruppe, er det nødvendig å inkludere 100 hoftebruddspasienter. En endring i ganghastighet på 0.1 m/s defineres som en klinisk relevant endring hos pasienter med hoftebrudd (10). En nylig meta-analyse på effekt av progressiv styrketrening hos eldre som inkluderte 24 studier med til sammen 1179 deltagere viste en gjennomsnittelig økning i ganghastighet på 0.08 m/s (11). Det er ikke urimelig å forvente en større effekt ved en intervensjon som har et mer spesifikt fokus på gange slik det legges opp til i dette prosjektet.

Observasjonsstudien viste at det er realistisk å inkludere tilnærmet 200 pasienter i løpet av 12 måneder med aktuelle inklusjonskriterier. Foreløpige gangdata fra Hip-Unit studien viser at cirka 2/3 lar seg teste for gange 4 måneder etter bruddet, mens cirka halvparten har gangdata både ved 4 og 12 måneder. Ved å planlegge en inklusjonsperiode på cirka 1 år vil cirka 135 pasienter være tilgjengelig for testing 4 måneder etter bruddet og cirka 100 ved 12 måneder.

Resultatmål

Funksjon før operasjon kartlegges retrospektivt ved hjelp av spørreskjema for personlig og instrumentell ADL (12, 13) og kognitiv funksjon (14) til pårørende/pasient. Etter 4 måneder benyttes de samme målene i tillegg til en funksjonstest for fysisk funksjon; Short Physical Performance Battery (15;16), et spørreskjema om bekymring for å falle; Fall Efficacy Scale

International (17), et spørreskjema om depresjon; Geriatric Depression Scale (18), et spørreskjema om helserelatert livskvalitet og mobilitet: EuroQual-5 (19), og en test på global kognitiv funksjon; Mini Mental Status Examination (20). Opplysninger om bruddtype og operasjonsmetode, sykdommer, medikamenter og komplikasjoner etter operasjonen innhentes fra pasientjournal.

Balansekontroll under gange måles ved hjelp av akselerometri (tre-akset akselerasjonssensor, TRASK)(21). Spatiotemporale gangparametre måles ved bruk av eletronisk gangmatte (GaitRite). En aktivitetsmåler, ActivPAL festes på forsiden av låret og benyttes til å registrere fysisk aktivitet gjennom 6 dager 4, 6 og 12 måneder etter operasjon. Variabler utledet fra aktivitetsregistreringene er gjennomsnittlig tid i oppreist stilling, antall skritt og antall forflytninger samt antall og lengde på aktive perioder.

Etiske betraktninger

Det vil bli innhentet informert skriftlig samtykke fra alle deltakerne. Mange pasienter har sannsynligvis redusert almenntilstand, eventuelt med smerte og forvirring, når de ankommer sykehuset og rett etter operasjonen og det kan da være vanskelig å gi samtykke til å delta i en slik studie. Dette gjelder også for deltakere som har nedsatt kognitiv funksjon. Pårørende kan i slike tilfeller reservere mot deltakelse så lenge pasienten selv er ute av stand til å gi eget samtykke. Pasienter som inkluderes i slike tilfeller vil få informasjon på nytt dersom tilstanden bedrer seg, og har da anledning til å trekke seg fra studien og få data slettet. Deltakerne rekrutteres til deltakelse i begge delstudiene av Eva-Hip under sykehusoppholdet. Deltakerne vil likevel på nytt bli forespurt om de fortsatt ønsker å delta i treningsstudien fire måneder etter bruddet.

Formidling

Det planlegges å publisere minst 4 artikler fra prosjektet i internasjonale tidsskrift innen klinisk medisin og geriatri. Resultatene vil bli presentert på nasjonale og internasjonale konferanser. I tillegg til planlagte vitenskapelige publiseringer innbefatter prosjektet også et fagutviklingsprogram rettet mot rehabiliteringstjenste og formidling av erfaringer fra dette til andre kommuner til andre kommuner gjøres på forespørsel. Resultater fra prosjektet vil også bli formidlet gjennom populærvitenskapelige artikler i norske tidsskrift, og gjennom foredrag til pasientorganisasjoner og helsepersonell.

Fremdriftsplan

En vesentlig del av prosjektet er utarbeiding av intervensjonen i både forløps- og treningsstudien. Mest tid vil bli satt av til dette i begynnelsen av prosjektet. Det vil også være nødvendig med oppfølging av fysioterapeutene som gjennomfører intervensjonen gjennom hele prosjektperioden. Inklusjon av pasienter til forløpsstudien kan starte så snart inklusjon i den pågående Hip-Unit-studien er avsluttet, og etter planen i februar 2011. Randomisering til treningsstudien skjer 4 måneder i etterkant av inklusjon. For å oppnå utvalgsstørrelsen vi trenger vil inklusjonsperioden bli på cirka ett år, men ved større frafall enn forventet må inklusjonsperioden forlenges.

References

(1) Hektoen LF, Aas E, Lurås H. (2009) Cost-effectiveness in fall prevention for older women. Scand J Public Health. 2009 Aug;37(6):584-9.

(2) Abrahamsen B, van ST, Ariely R, Olson M, Cooper C. Excess mortality following hip fracture: a systematic epidemiological review. Osteoporos Int 2009 October;20(10):1633-50.

(3) Chudyk AM, Jutai JW, Petrella RJ, Speechley M. Systematic review of hip fracture rehabilitation practices in the elderly. Arch Phys Med Rehabil 2009 February;90(2):246-62.

(4) Lloyd BD, Williamson DA, Singh NA, Hansen RD, Diamond TH, Finnegan TP et al. Recurrent and injurious falls in the year following hip fracture: a prospective study of incidence and risk factors from the Sarcopenia and Hip Fracture study 1. J Gerontol A Biol Sci Med Sci 2009 May;64(5):599-609.

(5) Solberg H, og medarbeidere. Artikkel under utarbeiding.

(6) Saltvedt I. Behandling av akutt syke, skrøpelige eldre pasienter i en geriatrisk specialavdeling. NTNU 2006.

(7) Helbostad JL. Motor functioning in community-dwelling older adults. Assessment and intervention. UIB 2004. ISBN: 82-7788-150-9

(8) Binder EF, Brown M, Sinacore DR, Steger-May K, Yarasheski KE, Schechtman KB. Effects of extended outpatient rehabilitation after hip fracture: a randomized controlled trial. *JAMA* 2004 Aug 18;292(7):837-46.

(9) Bellan van KG, Rolland Y, Bergman H, Morley JE, Kritchevsky SB, Vellas B. The I.A.N.A Task Force on frailty assessment of older people in clinical practice. J Nutr Health Aging 2008 January;12(1):29-37.

(10) Palombaro KM, Craik RL, Mangione KK, Tomlinson JD. Determining meaningful changes in gait speed after hip fracture. Phys Ther 2006 June;86(6):809-16.

(11) Liu CJ, Latham N. Can progressive resistance strength training reduce physical disability in older adults? A meta-analysis study. Disabil Rehabil 2010 May 17.

(12) Mahoney FI, Barthel DW. Functional evaluation: The Barthel Index. *Md State Med J* 1965 Feb;14:61-65.

(13) Gladman JR, Lincoln NB, Adams SA. Use of the extended ADL scale with stroke patients. *Age Ageing* 1993 Nov;22(6):419-424.

(14) Hughes et al 1982

(15) Guralnik JM, Winograd CH. Physical performance measures in the assessment of older persons [editorial]. Aging Milano 1994 October;6(5):303-5

(16) Guralnik JM, Ferrucci L, Pieper CF, et al. Lower extremity function and subsequent disability: consistency across studies, predictive models, and value of gait speed alone compared with the short physical performance battery. *J Gerontol A Biol Sci Med Sci* 2000 Apr;55(4):M221-M231.

(17) Helbostad JL, Taraldsen K, Granbo R, Yardley L, Todd CJ, Sletvold O. Validation of the Falls Efficacy Scale-International in fall-prone older persons. *Age Ageing* 2010 Mar;39(2):259. Epub 2009 Dec 22.

(18) Yesavage J, Brink T, Rose T, et al. Development and validation of a geriatric depression scale: a preliminary report. 1983. J Psychiatr Res 17: 37–49.

(19) Brooks R. EuroQol: the current state of play. Health Policy 1996 July;37(1):53-72.

(20) Folstein MF, Folstein SE, McHugh PR. "Mini-mental state". A practical method for grading the cognitive state of patients for the clinician. *J Psychiatr Res* 1975 Nov;12(3):189-198.

(21) Moe-Nilssen R, Helbostad JL. Modulation of gait during visual adaptation to dark. J Mot Behav. 2006 Mar;38(2):118-25.
