# Supplementary material for: Short and long-term clinical effectiveness and cost-effectiveness of a late-phase community-based balance and gait exercise program following hip fracture. The EVA-Hip Randomised Controlled Trial
Source: PLoS One. 2019 Nov 18;14(11):e0224971. doi: 10.1371/journal.pone.0224971 (PMC6860934; doi:10.1371/journal.pone.0224971)
Supplement: S2 File — (PDF) [file pone.0224971.s009.pdf]

Prosjektbeskrivelse EVA-HIP-studien

Evaluation of rehabilitation after hip fracture

Effekt av rehabiliteringstiltak på gangfunksjon og aktivitetsnivå etter hoftebrudd

1. Introduction

Norway has the world's highest incidence of hip fractures with just under 10,000 cases each year. A hip fracture is estimated to cost between 250,000 and 1.1 million kr the first year (1). A more effective rehabilitation offer would probably give a great socio-economic benefit. Hip fractures affect mainly elderly people, and hip fracture patients’ situation is usually complex. A hip fracture is associated with a significant reduction in functional and activity levels, increased mortality and increased need for help. During the first year after the fracture, 30% of the patients will be dead (2;3), and 20% of the patients who were home dwelling before the fracture will stay in a nursing home. More than half will have experienced new falls, and 30% have suffered injuries due to new falls (4).

An observational study conducted by the Research Group for Geriatrics (FFG) in 2007/2008 shows that the majority of elderly patients with hip fractures have significantly increased need for help and reduced functional levels after the fracture (5). This also applies to patients with a relatively high level of functioning before the fracture. This complies with other documentation and shows that a hip fracture is a dramatic event with major consequences for the affected person. It is also well documented that gait function is significantly impaired after a hip fracture. This is most likely an important cause of increased need for help and increased tendency to fall after the fracture. At the same time, several studies show that mobility and gait function can be improved as a result of targeted interventions (3), indicating a potential for achieving better gait function than is the case today.

Physical activity is probably also important for the recovery of physical function after a hip fracture. Nevertheless, we have limited knowledge about physical activity in hip fracture patients during the rehabilitation period, as well as how much dose and intensity of physical activity that is required to regain pre-fracture function. Since most people do not regain gait function, one can assume that the total amount of physical activity is too small. Today small body-worn activity sensors can be used to measure physical activity in everyday life over

several days. This type of information will be helpful to gain more knowledge of factors that can improve the effect of rehabilitation after hip fracture.

Experiences and feedback from patients who have participated in the above mentioned observational study and patients participating in an ongoing clinical controlled randomized study (Hip-Unit study) at St.Olavs Hospital and NTNU may indicate that the rehabilitation offer and follow-up after the fracture is fragmented and little adapted to the individual.

In order to ensure a good and comprehensive follow-up through the patient course, from hospitalization to rehabilitation is ended, it is necessary to focus on interaction between the various services and management levels. The action chain includes transition phases where the patient is moved from hospital, to rehabilitation facilities or directly to the home. The aim of this study is to ensure that elderly patients with hip fractures receive comprehensive and individually adapted follow-up throughout the rehabilitation course, to ensure the best possible recovery of gait function. To achieve this, the study will focus on services based on individual needs and with particular emphasis on physical activity.

2. Research group

Research group for geriatrics (FFG) is an interdisciplinary group of 13 people, three of which are senior researchers; Olav Sletvold, Jorunn Lægdheim Helbostad and Ingvild Saltvedt and 11 have master's degrees or higher. FFG has particular experience with clinically controlled and randomized trials. First major study compared the usefulness of treating frail geriatric patients in a specialized geriatric department with an internal department at St. Olavs Hospital, see Ingvild Saltvedt's doctoral thesis (NTNU 2002) (6). The implementation of this project was supported by the Ministry of Social Affairs and Health and the Research Council of Norway. The next study compared different types of home and home-grown training in Trondheim, see Jorunn L. Helbostad's doctoral dissertation (UiB 2004) (7).

Since 2005, FGG has focused especially on elderly people with gait difficulties, falls and fall injuries, especially hip fracture patients. The project "Assessment and treatment of older people at risk of falling" (2006-2008), which included for example an observational study of hip fracture patients 3 months after surgery, was supported by NOK 2 million from the Research Council of Norway. The same project also evaluated the usefulness of cataract

operations with regard to gait function and falls. In 2008, FFG received an NFR grant of NOK 3 million to carry out a project comparing traditional treatment of hip fracture patients in an orthopedic department against an orthogeriatric department. The possibilities for implementing the EVA-HIP project according to plan have been further strengthened in 2010 after the Collaboration Agency's grant of infrastructure resources for Ganglab of NOK 7.5 million over a 5-year period and that INM, DMF, NTNU has defined "Motion-Related Conditions "as a priority area of focus at the department.

The Eva-Hip study is conducted in close collaboration with the health service in Trondheim municipality, by municipal councilor Helge Garåsen. Head of unit for Physical Therapy in Trondheim Municipality Anne E. Hansen and Senior Coordinator for Older Sylvi Sand, and Randi Granbo (HIST, Coordinator of Trondheim Municipality) will be important partners in relation to the development of service provision in such a rehabilitation chain and implementation of the intervention in municipality.

3. AIM and research questions

The overall purpose of the Eva-Hip study is to improve the rehabilitation offer for older people after hip fracture after hospitalization. The project consists of two studies.
Part 1 includes the trajectory from discharge to four months after the fracture, and part 2 is an exercise trial starting four months after hip fracture. In the following, the two sub-studies are referred to as the course study and the exercise study, respectively.

The course study aims to see whether an extended comprehensive patient course can affect the activity level and gait function by introducing fixed time for functional assessments after hospitalization and increasing knowledge about and focusing on physical activity and recovery of gait function. The exercise study aims to increase the gait function and activity level after hip fractures through a specific exercise program offered 4-6 months after the fracture. The goal is to investigate the specific exercise effect on the activity level and gait function after hip fracture.

Research questions:

1. What is the effect of a new and comprehensive patient course focusing on rehabilitation as compared to usual care after hospitalization, on the amount and type of physical activity?
2. What is the effect of a new and comprehensive patient course focusing on rehabilitation compared to usual care after hospitalization, on gait function?
3. What is the effect of an individually tailoried strength and balance exercise program conducted 4-6 months after hip fracture as compared to usual care, on pysical activity and gait function?

4. Methods

Target population

The target population for this study is elderly hip fracture patients ≥ 70 years who were home dwelling with ability to walk (10 meters with or without aids) prior to the hip fracture. Persons resident in the municipality of Trondheim and admitted to St. Olavs Hospital with hip fractures in the upper femur (medial, per- and subtrochanteric) are included. Patients with a short life expectancy (<3 months), multi-trauma or pathological fractures are excluded. Cognitive decline is not an exclusion criterion, as cognitive impairment is a group that is particularly interested for the evaluation of rehabilitation efforts. For the exercise study, patients who cannot walk 10 meters 4 months after the fracture or have any medical conditions where exercise is contra-indicated are excluded from participation.

Design

The project consists of two sub-studies; the course study and the exercise study. The course study focuses on the patient course during the first four months after the break and evaluates an extended comprehensive patient course with fixed time for functional assessments after hospitalization. This study is conducted as a quasi-experimental cohort study with historical controls from the current Hip-Unit study. One hundred and thirty new patients are included in the casus group.

Upon completion of the follow-up four months after the fracture, the exercise study is introduced, where the participants from the study program are randomized to physical exercise or usual follow-up. This is thought to be a clinical randomized study, with parallel

group design, where exericse is compared to usal follow-up. Stratified block randomization is planned on the operation method and use of aids prior to the break (Yes / No). Assessors in the project will be blinded to the participants' group allocation. Randomization takes place through a web-based program under the auspices of the Unit for Applied Clinical Research at NTNU.

Procedures

Figure 1 shows the flow of participants in the Eva-Hip study, both the course study and the exercise study. Patient / operating lists at the Orthopedic Department and the Department of Geriatrics at St. Olavs Hospital are reviewed to identify participants for the study. The potential hip fracture patients are requested by hospital staff for participation in the study 3-7 days after surgery. Project registrations are completed by inclusion (during the index stay), including information from patient records and information obtained through structured interviews with patients / relatives. Four months after the hip fracture, the course study ends, and participants perform activity and function registrations. Participants who meet inclusion criteria for the exercise study will then be randomized to the exercise or control group, where the exercise group performs specific exercise twice a week for 10 weeks while the control group receives follow-up as usual. Twelve months after the break, activity and function registrations are carried out.

The project registrations will take place at the gait lab at the Department of Neuromedicine, DMF, NTNU. If this is not possible, we will use of a reduced test battery at patient home (institution or at home). Activity sensors are carried on the body over 6 days in the patient's habitat and collected by project staff.

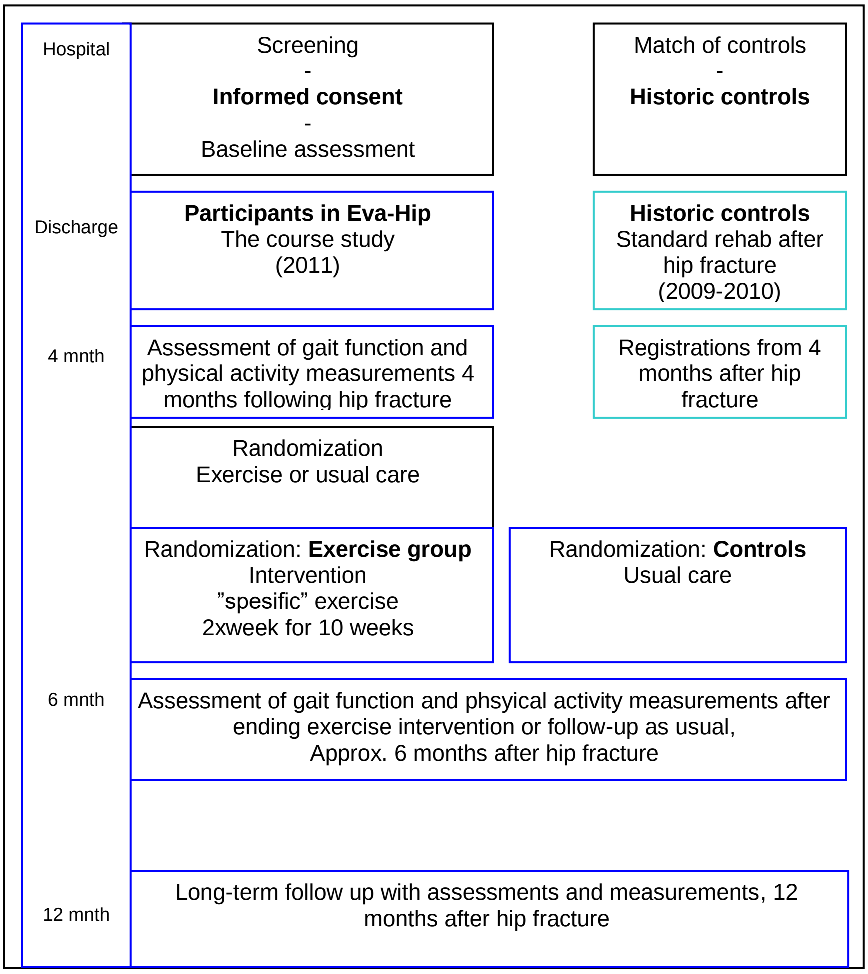

Figur 1. Flow of participants in the Eva-Hip study

Intervention

The course study using «early warning», a 3-day visit, 4-week assessment

The course study is anchored in the organizational structure now established as part of the service offer in Trondheim municipality through the "Overall Patient Progress" (HPH). HPH is the care and care services tool to ensure an optimal patient course for people who are being discharged to their homes and which will be "widened" in all municipal districts by December 2010. Many hip fracture patients will not be covered by HPH because they do not have any services from the municipality. This applies, for example, to those who only have practical help or security alarm, those who are on rehabilitation stays before returning home or those who travel directly to the home and who are not registered as healthcare users. In order to ensure an optimal patient course for all hip fracture patients in the Eva-Hip project, the local physiotherapists will have a special responsibility for using the HPH permanent assessment function tool at fixed time points throughout the trajectory after hip fracture.

In order for the physiotherapy service in the municipality to be able to perform early functional assessments for hip fracture patients after hospitalization, a new early warning system from the hospital will be established. This routine means sending a message to the referring physiotherapist about participants included in the project. After the referral responsible physiotherapist in the correct district has been notified that a patient is included and participates in the study, this will be registered as an active user. The municipal physiotherapist receives the first update on the patient by reading the note from the assessment meeting at the hospital and checking the discharge plan at the same time. Most hip fracture patients are likely to have a short-term stay at a health care or rehabilitation institution before returning home, but some travel directly home after hospital stay and others are accommodated in a nursing home. Expected time of returning from a rehabilitation institution or nursing home is obtained by the responsible physiotherapist in the municipality, and a functional assessment will be carried out 3 days after discharge to home so any services needed can be started early. The municipal physiotherapist then conducts a 4-week call to follow up and evaluate further offers. Participants in the project who stay at a nursing home will receive the same follow-up with assessment at 3 days and at 4 weeks.

In order to increase the knowledge about hip fracture in physiotherapists who will be in contact with hip fracture patients throughout the patient course, prior to the start of the project we will carry out seminars for physiotherapists where we focus on rehabilitation after hip fracture. The aim is to focus on interaction and exchange experience between physiotherapists working with the patient group at different levels of the service. The seminars will involve physiotherapists in hospitals, rehabilitation, health centers, private institutes and physiotherapists in the municipality.

The exercise study

The second part of the project consists of a physiotherapy intervention that takes place in close cooperation with the municipality's physiotherapy and physical institutions in the municipality. The intervention consists of a short and intensive exercise program x 2 per week over 10 weeks performed by physiotherapists in the Unit for Physical Therapy, Trondheim Municipality, or at a physical institute in the municipality, 4-6 months after the hip fracture. The training is individual and focuses on physical activity level and gait function. The specific content is planned and developed in collaboration with physiotherapists in Trondheim

municipality as a service development project. Potential physiotherapists who will take part in the development of the exercise program will be recruited during the seminars that we will have prior to project start in Eva-Hip.

Sample size and power calculation

Sample size and power calculation in this study are based on data from the observational study and the Hip-Unit study already completed. The patient group of hip fractures has high morbidity and mortality, and dropout will therefore be a particular challenge in this study. Other training in hip fracture patients has reported up to 30% drop in the intervention period (8).

There is broad agreement in the literature that walking speed is one of the best available measure for health and function in frail elderly people (9). The material from the observational study showed average gait speed 3 months after the break of 0.5 m/s with standard deviation of 0.2 m/s at preferred walking speed. With 90% power and 5% significance level, and a difference of 0.13 m/s in walking speed between control and intervention group, it is necessary to include 100 hip fracture patients. A change in walking speed of 0.1 m/s is defined as clinically meaningful in hip fracture patients (10). A recent meta-analysis of the effect of progressive strength training in the elderly that included 24 studies with a total of 1179 participants showed an average increase in walking speed of 0.08 m/s (11). It is not unreasonable to expect a greater effect of an intervention that has a more specific focus on gait that are added to this project.

The observational study showed that it is realistic to include almost 200 patients over 12 months with our inclusion criteria. Preliminary data from the Hip-Unit study show that approximately 2/3 can be tested for a period of 4 months after the fracture, while approximately half have gait assessments at both 4 and 12 months. By planning an inclusion period of approximately 1 year, approximately 135 patients will be available for testing 4 months after the fracture and approximately 100 by 12 months.

Outcome measures

Pre-fracture function is retrospectively collected by use of the personal and instrumental ADL (12, 13) and cognitive function (14) questionnaires for relatives / patients. At 4 months, the same measures are used in addition to a test of physical function; Short Physical Performance Battery (15; 16), a questionnaire about fear of falling; Fall Efficacy Scale International (17), a questionnaire about depression; Geriatric Depression Scale (18), a questionnaire about health-related quality of life and mobility: EuroQual-5 (19), and a test on global cognitive function; Mini Mental Status Examination (20). Information about fracture type and surgery, illnesses, medications and complications after surgery is obtained from patient records.

Balance control during walking is measured by accelerometers (three-axis acceleration sensor, TRASK) (21). Spatial temporal gait parameters are measured using an eletronic gait mat (GaitRite). An activity sensor, ActivPAL is attached to the front of the thigh and is used to record physical activity through 6 days 4, 6 and 12 months after surgery. Variables derived from activity registrations are the average time in upright position, number of steps and number of sit-to-stand transfers, as well as the number and duration of active periods.

Ethical considerations

Informed written consent will be obtained from all participants. Many patients have probably reduced general condition, possibly with pain and confusion, when they arrive at the hospital and immediately after surgery, and it may be difficult to give consent to participate in such a study. This also applies to participants who have reduced cognitive function. Caregivers may, in such cases, reserve for participation as long as the patient is unable to give his/her consent. Patients included in such cases will receive information again if the condition improves and may then withdraw from the study and get data deleted. Participants are recruited for participation in both studies of Eva-Hip during the hospital stay. Participants will nevertheless be re-asked if they still wish to attend the exercise study four months after hip fracture.

Dissemination

It is planned to publish at least 4 articles from the project in the International Journal of Clinical Medicine and Geriatrics. The results will be presented at national and international conferences. In addition to planned scientific publications, the project also includes a professional development program aimed at rehabilitation services and dissemination of experience from this to other municipalities to other municipalities made on request. Results

from the project will also be disseminated through popular science articles in norwegian journals, and through lectures to patient organizations and health professionals.

Work schedule

An important part of the project is the preparation of the intervention in both the course and the exercise study. Most time will be devoted to this at the beginning of the project. There will also be a need for follow-up of the physiotherapists who carry out the intervention throughout the project period. Inclusion of patients to the study program can start as soon as inclusion in the ongoing Hip-Unit study has been completed, and according to plan in February 2011. Randomization to the training program takes place 4 months after inclusion. In order to achieve the sample size we need, the inclusion period will last for approximately one year, but with a greater drop than expected, the period of inclusion must be extended.

References

(1) Hektoen LF, Aas E, Lurås H. (2009) Cost-effectiveness in fall prevention for older women. Scand J Public Health. 2009 Aug;37(6):584-9.

(2) Abrahamsen B, van ST, Ariely R, Olson M, Cooper C. Excess mortality following hip fracture: a systematic epidemiological review. Osteoporos Int 2009 October;20(10):1633-50.

(3) Chudyk AM, Jutai JW, Petrella RJ, Speechley M. Systematic review of hip fracture rehabilitation practices in the elderly. Arch Phys Med Rehabil 2009 February;90(2):246-62.

(4) Lloyd BD, Williamson DA, Singh NA, Hansen RD, Diamond TH, Finnegan TP et al. Recurrent and injurious falls in the year following hip fracture: a prospective study of incidence and risk factors from the Sarcopenia and Hip Fracture study 1. J Gerontol A Biol Sci Med Sci 2009 May;64(5):599-609.

(5) Solberg H, og medarbeidere. Artikkel under utarbeiding.

(6) Saltvedt I. Behandling av akutt syke, skrøpelige eldre pasienter i en geriatrisk specialavdeling. NTNU 2006.

(7) Helbostad JL. Motor functioning in community-dwelling older adults. Assessment and intervention. UIB 2004. ISBN: 82-7788-150-9

(8) Binder EF, Brown M, Sinacore DR, Steger-May K, Yarasheski KE, Schechtman KB. Effects of extended outpatient rehabilitation after hip fracture: a randomized controlled trial. *JAMA* 2004 Aug 18;292(7):837-46.

(9) Bellan van KG, Rolland Y, Bergman H, Morley JE, Kritchevsky SB, Vellas B. The I.A.N.A Task Force on frailty assessment of older people in clinical practice. J Nutr Health Aging 2008 January;12(1):29-37.

(10) Palombaro KM, Craik RL, Mangione KK, Tomlinson JD. Determining meaningful changes in gait speed after hip fracture. Phys Ther 2006 June;86(6):809-16.

(11) Liu CJ, Latham N. Can progressive resistance strength training reduce physical disability in older adults? A meta-analysis study. Disabil Rehabil 2010 May 17.

(12) Mahoney FI, Barthel DW. Functional evaluation: The Barthel Index. *Md State Med J* 1965 Feb;14:61-65.

(13) Gladman JR, Lincoln NB, Adams SA. Use of the extended ADL scale with stroke patients. *Age Ageing* 1993 Nov;22(6):419-424.

(14) Hughes et al 1982

(15) Guralnik JM, Winograd CH. Physical performance measures in the assessment of older persons [editorial]. Aging Milano 1994 October;6(5):303-5

(16) Guralnik JM, Ferrucci L, Pieper CF, et al. Lower extremity function and subsequent disability: consistency across studies, predictive models, and value of gait speed alone compared with the short physical performance battery. *J Gerontol A Biol Sci Med Sci* 2000 Apr;55(4):M221-M231.

(17) Helbostad JL, Taraldsen K, Granbo R, Yardley L, Todd CJ, Sletvold O. Validation of the Falls Efficacy Scale-International in fall-prone older persons. *Age Ageing* 2010 Mar;39(2):259. Epub 2009 Dec 22.

(18) Yesavage J, Brink T, Rose T, et al. Development and validation of a geriatric depression scale: a preliminary report. 1983. J Psychiatr Res 17: 37–49.

(19) Brooks R. EuroQol: the current state of play. Health Policy 1996 July;37(1):53-72.

(20) Folstein MF, Folstein SE, McHugh PR. "Mini-mental state". A practical method for grading the cognitive state of patients for the clinician. *J Psychiatr Res* 1975 Nov;12(3):189-198.

(21) Moe-Nilssen R, Helbostad JL. Modulation of gait during visual adaptation to dark. J Mot Behav. 2006 Mar;38(2):118-25.
