# Supplementary material for: Short and long-term clinical effectiveness and cost-effectiveness of a late-phase community-based balance and gait exercise program following hip fracture. The EVA-Hip Randomised Controlled Trial
Source: PLoS One. 2019 Nov 18;14(11):e0224971. doi: 10.1371/journal.pone.0224971 (PMC6860934; doi:10.1371/journal.pone.0224971)
Supplement: S3 File — (PDF) [file pone.0224971.s010.pdf]

Statistical analysis plan

The Eva-Hip Study

"Task specific exercise four months after hip-fracture"

Completed 22.06.2015

Chief Investigator: Olav Sletvold

Co-investigators: Jorunn Helbostad

Pernille Thingstad

Kristin Taraldsen

Members of the trial management group:

Olav Sletvold, Jorunn Helbostad, Pernille Thingstad, Kristin Taraldsen, Sylvi Sand, Randi Granbo, Sallie Lamb, Thorlene Egerton, Vidar Halsteinli

Independent reviewers: University of Oxford, Clinical trial unit

SAP Authors: Kristin Taraldsen, Pernille Thingstad, Vidar Halsteinli, Thorlene Egerton, Sallie Lamb & Jorunn Helbostad

1. Background

The aim of this RCT was to investigate the effectiveness of an extended, home based exercise program, on gait and activity in elderly with hip fractures. Different aspects of effectiveness will be evaluated according to the following aims: 1) generalizability/acceptability of the exercise program to the target population; 2) effectiveness of the intervention on gait and activity; 3) cost-effectiveness of the intervention.

The target population was older hip fracture patients, inhabitants of the municipality of Trondheim and community dwelling at time of the fracture. The intervention was a physiotherapy led, task specific, home based, exercise program initiated four months after hip fracture surgery. Participants received a total of 20 sessions, 2 sessions a week during 10 weeks. The study is a two-armed pragmatic, single-blind, stratified and random block-randomized controlled trial (RCT) with even distribution of patients in each arm. Power calculations indicated 220 participants needed to be included during the hospital stay to be able to detect a clinical meaningful difference of 0.1 m/sec in gait speed 12 months following the fracture. These calculations were based on drop-out and mortality rates found in former studies including the same population.

Patients were recruited during the hospital stay and randomized for exercise four months following the fracture. This run-in period allowed for collection of pre-fracture function and baseline characteristics on participants who were reluctant to participate in the exercise program but willing to attend the assessments. This design was chosen as it was considered important to be able to describe which patients found the program unacceptable. Inclusion and collection of pre-fracture information (T0) was carried out for participants shortly after the hip fracture surgery, either at the hospital or by telephone if discharge from hospital was early. Baseline assessment (T1) and randomization was scheduled four months (16 weeks) following the operation, follow-up (T2) 28 weeks after operation and (T3) 52 weeks after operation.

The inclusion period during hospital stay lasted from 18th of February 2011 to 27th of February 2013. The registrations at four months started the 16th of February 2011 and the last patient was tested the 1st of July 2013. Post-test started the 15th of September 2011 and ended the 3rd of October 2013. Follow-up at 12 months started the 12th of March and the very last day of follow up in this study was 3rd of March 2014.

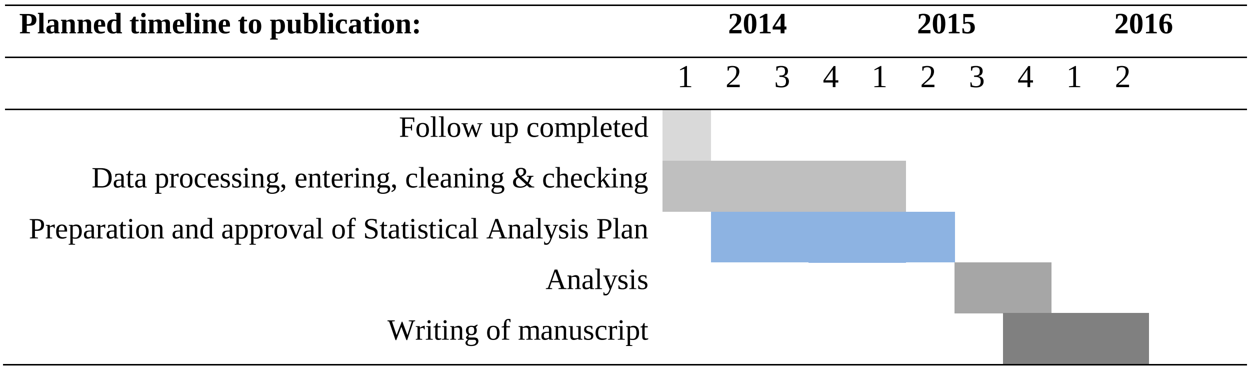

822 hip- fracture patients were screened for eligibility. 524 were not in the target population, either living outside the catchment area, being nursing home residents, younger than 70 years or not having a hip fracture. Among those in the target population 75% were recruited during the hospital stay. 28 were excluded from the run-in period because of medical contraindications (9,5%), 20 persons were participants in conflicting research projects (7%), 20 (7%) did not want to participate, and 7 (2%) were never asked to participate. From the recruiting period at the hospital and till randomization, 13 patients had died and 27 declined to participate further in the study. 183 participants completed the baseline assessment (T1) but 24 people declined to participate, 12 were excluded due to medical contraindications, and five were excluded due to being bedbound. Thus, 142 participants were randomized, of whom 123 were tested at T2, and 112 at T3.

2. Aims of the statistical analysis

Aim of this statistical analysis plan is to describe the analysis for the final reporting of the trial according to the following three aims of the trial:

1) Generalizability and acceptability: Describe to which extent the target population is reached, including reasons for dropouts and characteristics of those who refused to participate in the exercise program, but willing to attend assessments. The flowchart aim to provide a full overview of a complete cohort of hip-fracture patients operated within the inclusion period, including reasons for exclusions, withdrawal and drop-outs at T0, T1, T2 and T3.

2) Effectiveness of the intervention on clinical outcomes: Group differences in primary (gait speed and activity) and secondary outcomes (mobility, ADL, cognition, depression and falls efficacy) will be assessed at T2 and T3

3) Cost-effectiveness of the intervention: Assessment of group differences in health care utilization and costs for the different time periods T1-T2 and T2-T3, and specifically T1-T3. Assessment of group differences in quality adjusted life years for the period T1-T3. Analyze incremental costs and effects in order to assess the cost-effectiveness of the intervention; that is calculating the incremental cost effectiveness ratio (ICER).

3. Populations

Total population: Patients who were recruited during the hospital stay will be described using descriptive statistics, n=223

Intention to treat: The primary analysis will include all patients who were randomized at T1, n=142

Complier averaged causal effect analysis: We assume that one has to participate in the exercise intervention to benefit from it, and compliers were defined as participants within the treatment arm completing 15 (75%) of the sessions within 20 weeks (n=45). A complier averaged causal effect analysis will be performed.

4. Analysis datasets

Two datasets will be produced: 1) Observed dataset: quality checked of all data as observed at each visit; 2) Imputed dataset on single items. We will handle item level missing data at using published guidelines for the relevant test scores, or simple alternative if appropriate (see individual test scores in appendix).

Our initial analysis will use data on all complete cases with imputed single items, and a secondary sensitivity analysis of the compliers of the intervention.

We will investigate patterns of item and case level missing data using descriptive methods, focusing on baseline and recovery trajectories for those with case level missingness. We will consider the pattern of missing data (missing at random, missing completely at random and missing not at random), according to how we handle the missing data we will consider to do a sensitivity analysis by use of multiple imputation technique.

5. Protocol violators

Two types of protocol violators will be described:

Research protocol violation: Numbers and reasons will be presented related to

1) Missed routines

2) Technical problems with equipment.

Intervention protocol violation (compliance): Numbers and reasons for non-adherence will be presented (n=23) by treatment arm:

1) Hospitalized

2) Too burdensome

3) Vacations, out of town

4) Deceased

5) Pain

6) Illness

6. Withdrawals

Reasons for withdrawal at T1, T2 and T3: 1) Unable due to medical condition, hospitalization 2) Find it too burdensome. 3) Do not see the advantage of participating. 4) Moved permanently or temporarily outside catchment area.

7. Sample descriptors

- Demographics: Age, gender, living alone, Charlson comorbidity
- Fracture/surgery: Intra/extra capsular, osteosynthesis/arthroplasty
- Barthel Index, Nottingham Extended ADL Index, CDR, walking aids (indoor/outdoor), circumstances of hip fracture fall (location, time, reason)

8. Harms and significant adverse events

- Adverse events that occurred during the intervention and follow-up periods will be reported, including number of falls, decline in health, and death.
- Adverse events that occurred in relation to the exercise, and could be attributed to participation in the intervention, will be reported separately. These adverse events will include any reports of pain, falls, morbidity, mood disturbances, and decline in health.

9. Variables

9.1 Treatment descriptors (aim 1)

- Presentation of flow of participants in the trial (flow chart, see attachment)
- Additional descriptive information about the acceptability of the trial:
  - Number and proportion of completers
  - Number and proportion of complete sessions (all 5 exercises performed)
  - Number and proportion of participants at each level (1-5) for each of the five exercises the first and last session
  - Level of exercise fatigue/exhaustion (median and IQR)
- Description of the physiotherapists delivering the intervention (number and years of experience)

9.2 Clinical outcomes (aim 2)

- **Primary outcome:** Gait speed (m/s)
- **Secondary outcomes:**
  - Fast and dual task gait speed (m/s)
  - Preferred speed (m/s), temporal-spatial gait variables including mean and variability of step length (cm), step width (cm) and the proportion of time per gait cycle in single support (% time of one gait cycle), plus walk ratio (steplenght/cadence), asymmetry of step length, and asymmetry of single support (100 x (|ln(left/right)|)
  - Physical activity outcomes including mean and variability of upright and sedentary time and number of upright events
  - Mobility performance (SPPB (0-12) and TUG (seconds)
  - ADL (BI and Nottingham Extended I-ADL) (0-64)
  - Cognitive function (CDR scale (0-18) and MMSE (0-30))
  - Depression (GDS) (0-15)
  - Health-related quality of life (EQ-5D) (–0·594 to 1)
  - Short Falls efficacy Scale International, FES-I (7-28) and fear of falling numeric rating scale (0-10)
  - Fatigue CFS (physical and mental subscales) (0-100)
  - Isometric muscle strength (knee extensor (kg) and grip strength (kg))
  - Pain (Pain NRS and pain in operated limb scale) (0-10)
  - Use of walking aid (yes/no) and self-report change in walking ability

9.3 Data on health care services (aim 3)

Hospital services

Use of hospital services in the period T1 to T3:

- In-patient stays (no of days)
- Outpatient visits (numbers)

Primary health care services

Use of primary care services in the period T1 to T3 (numbers):

- Rehabilitation stays and hour based rehabilitation (including physiotherapists)

- Nursing home stays

- Home nursing care

- Home care services

- Visits to general practitioner

10. Data quality control

Complete data files were checked for errors by manually check of the data set, descriptive statistics and performing checks on outliers. All outlying values (greater than 2SD) were verified against source data to ensure that they had not been inappropriately entered. Outliers were corrected or removed from the data set if there was evidence that it was an error.

A random selection of 10 % participants test scores was checked against the source data to describe the error percentage in the scanning. If any systematic errors are identified, the entire data set will be checked against the source data. After this procedure, error percentages higher than 5% on each form, the entire data set for this form will be checked against the source data.

11. Statistical analysis

11.1 Sample Size estimation

Sample size was calculated from an estimated effect size of 1·0 point in mean SPPB score at 4 months after surgery. (1·0 point is regarded as a substantial meaningful change, and 0·5 points is a small meaningful change). We expected a reduction of 10% in participants resulting from death and a 10% dropout because of withdrawals during the run-in period the first 4 months after surgery. With an α value of 0·05, 304 patients were needed for 80%

power, but 380 patients were needed to allow for an estimated 20% dropout rate before inclusion at 4 months.

11.2 General principles of the analysis

The significance level for the statistical analysis was set at 0.05 and all statistical analyses were carried out as two-tailed. All tests will be carried out with IBM Statistics SPSS 21 software.

Prefracture and baseline characteristics are presented using descriptive statistics mean (SD) or median (IQR), number (%) as appropriate for the distribution.

Variables will be visually inspected by Q-Q-plots and transformation of variables with non-normal distributions will be performed when appropriate.

The number of participants in each group will be included in the analysis and presented in the Tables.

11.3 Generalizability and acceptability

The flowchart will provide an overview of the complete cohort of hip-fracture patients operated within the inclusion period, including reasons for exclusions, withdrawal and drop-outs at T0, T1, T2 and T3.

Descriptive statistics of the total sample included (n=223) (pre-fracture ADL function and pre-fracture cognitive function) will be presented.

Descriptive statistics of randomized participants (n=142), dead prior to baseline (n=13), declined to participate (n=51), and excluded from participation (n=17) will be presented in Table 1. Pre-fracture and baseline characteristics (N=223) and differences in pre-fracture and baseline characteristics among the randomized and the three other groups will be presented using descriptive statistics: mean, median, percentages, and differences between those randomized and those not included calculated with t-tests, Mann Whitney U-tests and Fisher exact tests.

Descriptive statistics of all subgroups of participants (randomized, withdrawals, dead, and dropouts) for T0, T1, T2 and T3 will be presented in Table 2. Relevant group differences will be analyzed by use of descriptive statistics: mean, median, percentages, and differences between the groups with t-tests, Mann Whitney U-tests and Fisher exact tests.

Differences between compliers and non-compliers of the intervention will be presented for T0, T1, T2 and T3 by use of descriptive statistics and t-tests in Table 3.

11.4 Effectiveness on clinical outcomes

Differences in pre-fracture function and baseline characteristics between treatment arms (N=142) will be presented using descriptive statistics and t-tests/Mann Whitney U-tests in Table 4.

Main analysis will be performed according to intention to treat (N=142) (White, Horton, Carpenter, & Pocock, 2011).

All outcomes will be compared between groups using mixed-effects linear regression model with 'group' as fixed effect, and age, gender and fracture type as random effects (Twisk & de Vente, 2008). The treatment effect (point estimate of difference) with 95% confidence intervals (CIs) will be reported when relevant and a two-sided p-values <0.05 will be considered statistically significant (Table 5).

11.5 Cost analysis

Intervention costs, hospital service costs and primary health care service costs will be calculated and aggregated into total health care costs. Group differences will be analyzed by use of descriptive statistics: mean and median, and differences between the groups with t-test and Mann Whitney U-tests (table 6).

11.6 Cost-effectiveness analysis

The different health states generated from the EQ-5D-3L will be assigned values from the UK time-trade-off tariff. Quality adjusted life years (QALYs) will be calculated with the area-under-the curve approach, with an assumption of a linear change in EQ-5D-3L values over time. Cost-effectiveness will be evaluated by calculating the difference in mean costs and dividing by the difference in mean QALYs. Uncertainty about the incremental cost-effectiveness ratio (ICER) will be estimated using bootstrapping methods.

11.7 Data will be presented on adverse events, harms, and adherence/compliance

All important adverse events or unintended effects will be described by use of descriptive statistics for each group (Table 8). Data on reported harms related to the intervention will be described individually.

12. Sensitivity Analysis

Exercise group members rarely attend all sessions as compared to control group members who can participate more easily. This will especially be a challenge in our trial, including frail older persons after hip fracture. A sensitivity analysis to evaluate the actual impact of participating in the exercise intervention will therefore be performed by use of a complier averaged causal effect analysis. Compliers of the exercise intervention are defined as participants within the treatment arm completing 15 (75%) of the sessions within 20 weeks (n=45), and a complier averaged causal effect analysis will be performed.

13. Dissemination

All results of the main study will be presented in a single manuscript, written in accordance with the CONSORT 2010 guidelines for reporting clinical trials, and submitted for publication in a peer reviewed international journal

Cross-sectional and longitudinal observational analyses and qualitative data will be reported separately.

14. SAP Amendments

10.10.2014 Original version. No amendments

15. Appendices

Appendix A. Copies of all forms used in collecting the data.

Appendix B. Draft of the flow chart, Tables, and overview of tests at the T0, T1, T2 and T3 examination.

16. References

Twisk, J. W., & de Vente, W. (2008). The analysis of randomised controlled trial data with more than one follow-up measurement. A comparison between different approaches. [Comparative Study]. *European Journal of Epidemiology, 23*(10), 655-660. doi: 10.1007/s10654-008-9279-6

White, I. R., Horton, N. J., Carpenter, J., & Pocock, S. J. (2011). Strategy for intention to treat analysis in randomised trials with missing outcome data. [Randomized Controlled Trial

Research Support, N.I.H., Extramural

Research Support, Non-U.S. Gov't]. *BMJ, 342*, d40. doi: 10.1136/bmj.d40
