# Supplementary material for: Short and long-term clinical effectiveness and cost-effectiveness of a late-phase community-based balance and gait exercise program following hip fracture. The EVA-Hip Randomised Controlled Trial
Source: PLoS One. 2019 Nov 18;14(11):e0224971. doi: 10.1371/journal.pone.0224971 (PMC6860934; doi:10.1371/journal.pone.0224971)
Supplement: S4 File — (PDF) [file pone.0224971.s011.pdf]

RESEARCH ARTICLE

Effectiveness of Task Specific Gait and Balance Exercise 4 Months After Hip Fracture: Protocol of a Randomized Controlled Trial — The Eva-Hip Study

Pernille Thingstad[1]*, Kristin Taraldsen[1], Gunhild Hagen[2], Sylvi Sand[3], Ingvild Saltvedt[1,4], Olav Sletvold[1,4] & Jorunn L. Helbostad[1,5]

[1]Department of Neuroscience, Norwegian University of Science and Technology, Trondheim, Norway

[2]Department of Public Health and Community Medicine, Norwegian University of Science and Technology, Trondheim, Norway

[3]Unit for Physiotherapy Services, the Municipality of Trondheim, Trondheim, Norway

[4]Department of Geriatrics, St Olav University Hospital, Trondheim, Norway

[5]Department of Clinical Services, St. Olav University Hospital, Trondheim, Norway

Abstract

Background and purpose. Regular rehabilitation is not sufficient for regaining function after a hip fracture, and more targeted interventions for home-dwelling elderly hip-fracture patients are needed. This paper describes the protocol of a study assessing the effectiveness and cost effectiveness of a task specific progressive gait and balance exercise programme for hip-fracture patients, performed 4 months after the fracture. **Methods/design.** A single blind two-arm pragmatic randomised controlled trial was conducted with 142 hip-fracture patients randomized to a 10-week home-based exercise programme or to practice as usual 4 months following the surgery. Inclusion criteria were age >70 years and being home dwelling prior to the fracture. Exclusion criteria are life expectancy <3 months and inability to walk 10 m prior to the fracture. The content and organization of the programme was developed in collaboration between physiotherapy researchers and primary health-care physiotherapists. Participants were followed for 1 year post-surgery, evaluating short-term and long-term effects of the programme. The primary outcome is gait speed, and the secondary outcomes are spatial and temporal gait parameters, free living physical behaviour by activity monitoring, mobility performance, activities of daily living, fear of falling, cognitive function, depression and health-related quality of life. Cost-effectiveness analysis is planned. **Discussion.** This paper describes a task specific exercise programme aimed to improve gait and balance after a hip fracture. Inclusion started in February 2011, and the last 1-year follow-up is performed in March 2014. Broad inclusion criteria and physiotherapy-guided home-based exercises may facilitate the participation from frail patients and thereby increase the generalizability of the findings. Development and completion of the intervention within routine clinical practice will enlighten the implementation of results into clinical practice. Results may add new insight into how physiotherapy can improve gait and thereby activity and functioning in everyday life and have implications on future content and organization of physiotherapy after a hip fracture. © 2014 The Authors. *Physiotheraphy Research International* published by John Wiley & Sons Ltd.

Received 29 November 2013; Revised 17 March 2014; Accepted 1 June 2014

Keywords

accelerometry; ageing; exercise; gait; hip fracture

***Correspondence**

Pernille Thingstad, Department of Neuroscience, Norwegian University of Science and Technology, Trondheim, Norway.

Email: pernille.thingstad@ntnu.no

Published online 3 July 2014 in Wiley Online Library (wileyonlinelibrary.com) DOI: 10.1002/pri.1599

This is an open access article under the terms of the Creative Commons Attribution License, which permits use, distribution and reproduction in any medium, provided the original work is properly cited.

Background

Hip fractures are associated with high age, frailty and permanent disability (Bertram *et al.*, 2011) including increased risk for new falls and fractures (Lloyd *et al.*, 2009), fear of falling, severely reduced quality of life (Ziden *et al.*, 2008b; Rohde *et al.*, 2010; Jellesmark *et al.*, 2012) and low levels of physical activity (Resnick *et al.*, 2011). Obtaining efficient and safe gait following a hip fracture could mean the difference between a home-dwelling, active and independent life and dependency and need for residential care. Despite evidence for the beneficial effect of early physiotherapy and exercise after hip fracture, there is insufficient evidence for best practice (Handoll *et al.*, 2011) and even less is known about the effect of extended exercise interventions and long-term effects of exercise interventions. The current knowledge base is mostly based on a few efficacy driven studies performed under highly controlled conditions or including participants that are relatively homogenous compared with the general home-dwelling hip fracture population (Orwig *et al.*, 2011; Sylliaas *et al.*, 2012; Latham *et al.*, 2014). It is not obvious that exercise programmes proven effective in efficacy studies will produce the same effect during real-world conditions (Flay *et al.*, 2005)

Earlier studies have usually evaluated the effect using mobility tests and self-reported measures of activity. To our knowledge, there are few studies that have evaluated the effect of exercise intervention on specific gait characteristics in combination with objective measures of activity after hip fracture. Such knowledge could be important to be able to develop more targeted and effective exercise programmes.

Gait and balance are the key aspects of mobility in daily life. It may be hypothesized that exercises for balance and gait especially for frail older persons with limited capacity will be more effective if they are performed under conditions similar to those encountered during daily life. Task specific exercises that aim to improve motor control represent a different approach from traditional exercise programmes, where strength, balance and endurance are trained as separate components (Sherrington and Henschke, 2013). This has been supported by VanSwearingen *et al.* (2011) who found that task specific exercises improved gait efficiency and activities of daily living (ADL) and increased the amount of physical activity in elderly people with impaired gait more than traditional impairment-oriented exercises (VanSwearingen *et al.*, 2011).

Basic mobility tasks of daily life involve frequent shifts between positions, such as sitting down or getting up, short walks, turns, stepping sideways or backwards or climbing stairs, all involving weight bearing over a changing base of support. Inadequate weight transfer, and reduced ability to adjust the body's centre of mass in relation to a changing base of support, is associated with balance impairments, falls (Robinovitch *et al.*, 2013) and hip fractures (Singer *et al.*, 2013; Winter, 1995). The association between impaired executive function, gait impairments and fall risk is well established, and there is increasing evidence for the effect of dual task training to improve gait and balance in elderly people with increased fall risk (Hsu *et al.*, 2012; Liu-Ambrose *et al.*, 2013; Montero-Odasso *et al.*, 2012).

Hip-fracture patients tend to be old and frail, and rehabilitation is often too short for gait performance to recover. Cognitive decline (Seitz *et al.*, 2011), depression (Holmes and House, 2000), fear of falling (Visschedijk *et al.*, 2010; Visschedijk *et al.*, 2013) and fatigue (Folden and Tappen, 2007) may restrict participation in community-based exercise programmes or even in home exercise programmes that are based on exercises that are not supervised. Patients that are vulnerable to deterioration in health and to functional decline may thus not receive sufficient follow-up after returning to their own homes, and may therefore not regain optimal functional abilities. A systematic review and meta-analysis suggested that exercise programmes initiated after the end of standard rehabilitation are promising strategies to improve independence in daily life activities after hip fracture but marked the lack of cost-effectiveness studies of extended programmes (Auais *et al.*, 2012).

This paper describes the protocol of a study that aims to evaluate the effectiveness and cost effectiveness of a task specific, home-based exercise programme initiated 4 months after the surgery. A specific focus has been on including a representative sample, to run the trial within real-world conditions and to include a broad spectrum of outcomes.

Methods

Participants were recruited between February 2011 and February 2013, at St Olav University Hospital, the regional hospital for the municipality of Trondheim (180,000 inhabitants and approximately 300 hip fractures

annually). Randomization and intervention were finished by June 2013, and the last follow-up will be performed in March 2014.

Context

The standard path for home-dwelling hip-fracture patients in Trondheim is to be transferred from the hospital to a rehabilitation facility within the first week after surgery. Time spent at the rehab facility varies from 2 to 8 weeks. The frailest and most dependent patients are discharged to nursing homes. A small number of patients are discharge directly to their own homes. There are no standards for content, intensity or length of physiotherapy offered to hip-fracture patients after they have returned home. Available services are home-based physiotherapy as a single service, physiotherapy as part of an ambulatory rehabilitation team or treatment in private physiotherapy clinics.

Overview of the study

This trial intends to evaluate the effectiveness of a late-phase exercise intervention. Participants were included within the first 5 days after the fracture, whereas baseline registrations and randomization took place 4 months following the fracture and the last assessment was 12 months after the fracture.

Design

The study is a two-arm pragmatic, single blind, block randomised controlled trial with even the distribution of patients in each arm.

Participants

The evaluation of eligibility was performed in two steps, first during the index stay and then as part of the baseline registrations at 4 months. Eligible participants were home dwelling prior to the fracture, lived in the municipality of Trondheim, were 70 years or older, diagnosed and underwent surgery for intra-capsular or extra-capsular hip fractures International Classification of Diseases (ICD-10 S72.0-S72.2)[1] resulting from a low trauma incident. Patients were excluded if the fracture was pathological, life expectancies were less than 3 months, they were unable to walk 10 m (with or without walking aids) prior to the fracture or were participating in one of two defined conflicting research projects. Patients who were bedridden or had medical contraindications for training as evaluated by a geriatrician at the time of baseline registration 4 months post-surgery were excluded before randomization.

Inclusion and randomization

Informed consent was obtained within the first 4 days after the surgery. For patients deemed non-competent in giving informed consent by a subjective evaluation by the case nurse, a next of kin was approached for a preliminary consent. After the completion of the baseline assessment at 4 months, participants were randomized to task specific exercise or usual care. The randomization was performed using a web-based computerized randomization service developed at the Unit for Applied Clinical Research, Norwegian University of Science and Technology. A stratified block randomization technique was used to ensure balanced group concerning intra-capsular versus extra-capsular fractures and pre-fracture use of walking aid (rollator indoor or not). All details concerning the solution were undisclosed to the research staff until end of inclusion. Group assignment through the web programme was performed by an administrative coordinator in the community health service who was not involved in any contact with participants. This person received identification number and name of participants for randomization from the research staff, ran the computer programme and noticed the physiotherapist who was to follow the patient.

Blinding

Assessors and personnel performing statistical analyses were blinded to participants' group allocation. Participants were instructed not to provide information that could reveal group allocation to the researchers or the assessors during the study period.

Intervention

The exercise programme was developed in collaboration between physiotherapy researchers and clinical physiotherapists working in home-based rehabilitation. By combining theoretical foundation with clinical expertise, we aimed at developing a task specific exercise programme that was standardized but could be tailored to individual needs and capabilities. The programme was intended to be feasible for routine clinical work.

[1]International Classification of Diseases, World Health Organization.

Patients randomized to the new exercise intervention received a home-based programme starting 4 months post-surgery, supervised by a physiotherapist twice weekly for 10 weeks, each session lasting approximately 45 minutes. The programme consisted of the following five weight-bearing exercises, all entailing change in base of support (Appendix): 1) walking; 2) stepping in a grid pattern; 3) stepping up on a box; 4) sit-to-stand; and 5) lunge. Each exercise is described at five difficulty levels to allow for the standardized registration of individualization and progression.

Progression was obtained by introducing variations in the task to challenge weight transfer, increasing movement speed, adding weight by using weight-vests, introducing more complex combinations of movements, and by adding secondary tasks (dual task condition). Exercises were meant to be performed without compensating strategies such as hand support or asymmetric weight bearing. Ten physiotherapists with varying background and experience were responsible for administering the exercise programme, as part of their ordinary work in the municipality.

Patients allocated to the control group received treatment as usual, which included a variety of different approaches, from no follow-up at all to quite extensive interdisciplinary rehabilitation in their homes or in an institution. Patients in the intervention group were given a choice whether to continue the treatment they already received in addition to the exercise programme they were randomized to, or to postpone this too after completing the exercise intervention.

Study assessments

Assessment was performed at four time points: (T1) during the hospital stay, (T2) 4 months post-surgery, (T3) within 2 weeks after conclusion of the intervention and (T4) 1 year post-surgery. During (T1) the hospital stay, only data on pre-fracture ADL and cognitive function were collected, whereas the full test battery, including 4 days activity monitoring, was performed at T2, T3 and T4. At T2, after baseline registrations, participants were randomized to take part in a 10-week home-based exercise programme or to receive usual care. Patients who were reluctant to participate in the exercise programme or dropped out during the follow-up period were still encouraged to meet for study assessments. All trial registrations were performed by experienced physiotherapists who were blind to group allocation and not involved in the exercise programme. Information on pre-fracture ADL and cognitive function collected at T1 were based on information from the patient, next of kin and medical record. Information on cognitive function was collected routinely from next of kin. (T2) Baseline, (T3) post-intervention and (T4) 1-year assessments were performed at the outpatient clinic and the movement laboratory at the hospital. Patients who were unable or reluctant to attend were offered a home visit with a modified protocol not including GAITRite® mat (CIR systems Inc. Sparta, US) or measures of knee extension muscle strength but otherwise the same battery. For the intervention group, level of progression, number of repetitions, time spent on each exercise and fatigability following each training session were reported on standardized forms.

Outcomes

The primary outcome measure is preferred gait speed. Secondary outcome measures are spatial and temporal gait parameters, physical activity, mobility performance, ADL, cognitive function, depression, health-related quality of life, falls efficacy, fatigue and fall rate during the follow-up period.

Preferred gait speed is regarded as a robust and sensitive measure of overall health and function and has been recommended as an outcome in interventions on elderly populations (Abellan van Kan *et al.*, 2009). Other outcomes were chosen to cover a broad spectrum of health-related aspects relevant in frail populations, and to include both performance-based measures of physical function, objective measures of free living physical behaviour and self-reported health. Outcomes commonly used within geriatric research were chosen for the purpose of comparison with other trials.

Gait variables were measured by means of an electronic walkway (GAITRite®) (Kressig *et al.*, 2006). Participants walked back and forth across a 10-m walkway, where the middle 4.88 m were recorded by the gait mat, in preferred, slow and fast self-administered speeds, and at preferred speed while counting backwards, for a total of eight walks. Gait variables include mean and variability of spatial variables, step length and step width and temporal variables, and the proportion of time per gait cycle in single support during preferred speed. Walk ratio is calculated as the ratio between step length and cadence at fast gait speed (Rota *et al.*, 2011). Dual task effects are expressed as

the percentage differences between single and dual task conditions (Plummer-D'Amato *et al.*, 2012). Asymmetry in step length and single support is calculated as the ratio between the affected and the non-affected leg during preferred speed (Yogev *et al.*, 2007).

Assessment of other outcomes

Basic and instrumental ADL (I-ADL) was measured by the Barthel Index (Mahoney and Barthel, 1965) and the Nottingham Extended I-ADL Scale (Nouri and Nb, 1987). Mobility was assessed by the Short Physical Performance Battery (Guralnik *et al.*, 1994) and the Timed Up-and-Go test (Podsiadlo and Richardson, 1991). Physical activity was measured by single-axis accelerometers over 4 days (activPALs from PAL Technologies ltd, Glasgow, UK), attached to participant's non-affected thigh (Taraldsen *et al.*, 2014). Outcome measures are mean upright time and mean number of upright events. Isometric knee extension strength was measured by a dynamometer (MIE limited edition, LTD) with the subject seated.

Health-related quality of life was measured using the EuroQol-5D-3L (Rabin and de Charro, 2001). The Mini Mental State Examination (Folstein *et al.*, 1975; Strobel and Engedal, 2008) and the Clinical Dementia Rating Scale (Hughes *et al.*, 1982) were used for the evaluation of cognitive status. Depression was assessed by the Geriatric Depression Scale (Sheikh and Yesavage, 1986) and falls efficacy by the 7-item Short Falls Efficacy Scale International (Hauer *et al.*, 2011; Helbostad *et al.*, 2010). The Chalder Fatigue Questionnaire (Chalder *et al.*, 1993) was used to assess chronic fatigue and an 11-point numeric scale to evaluate fatigability following each exercise session. The number of falls and fall circumstances during the follow-up period is registered on the basis of retrospective reports from participants, next of kin and physiotherapists. The outcome measures are the same as used in previous studies on hip-fracture patients from our research group (Sletvold *et al.*, 2011).

Utilization of health services

Costs will be calculated applying a broad health-care perspective. Data on use of hospital services (inpatient, day patient or outpatient services) and medications will be collected from the participants' hospital medical records. Data on use of health services delivered by the municipality units will be collected from the participants' municipality records, for example, home-based services and short-term nursing home stay. The use of services from general practitioners and private physiotherapists will be collected from the Norwegian Directorate of Health.

Sample size calculations and statistical analysis

Previous studies in otherwise healthy hip-fracture patients have reported meaningful difference in preferred gait speed in the range of 0.08 (Perera *et al.*, 2006) and 0.12 m/sec (Kwon *et al.*, 2009), whereas other studies suggest that meaningful change in gait speed is population dependent and probably higher for less healthy populations (Alley *et al.*, 2011). We expected our sample to be less healthy and with a larger within as well as between subject variance compared with the sample referred in the previous studies. Therefor a meaningful difference of 0.15 m/sec were selected for the power calculations. With a power of 90% and $p = 0.05$, a sample size of $n = 54$ in each arm are necessary to detect a difference in gait speed between groups of 0.15 m/sec. On the basis of the data from a previous study in hip-fracture patients with similar inclusion criteria performed by our research group, we expect about 20% to refuse to participate in the intervention, further 15% to have die during the follow-up period, 10% to be excluded because of medical contraindications or lost of ability to walk and about 10% to be expected to withdraw (Sletvold *et al.*, 2011). On the basis of these assumptions, we estimated the number of patients needed to be included during the index stay to be 220.

Statistical methods

All data will be analysed and presented according to the updated CONSORT guidelines for reporting parallel group trials including intention to treat and per-protocol analysis (Schulz *et al.*, 2010). Descriptive statistics will be used to describe patient characteristics and drop out during the follow-up period. As a first choice, primary and secondary outcomes will be analysed using the analysis of covariance (ANCOVA) (Vickers and Altman, 2001), adjusting for age, gender, pre-fracture I-ADL and baseline cognitive function (Mini Mental State Examination < 28).

Cost effectiveness will be analysed by calculating the incremental cost-effectiveness ratio (ICER) as the difference in costs between the two treatment groups, divided by their difference in effects measured as the quality-adjusted life years. We will use the time trade off tariff values from the United Kingdom to value the EQ-5D-3L health states (Dolan, 1997). Where there are incomplete benefits or cost data due to loss to follow-up, we will use non-parametric methods to infer cumulative costs and benefits. To derive confidence intervals for the ICER, non-parametric bootstrap methods will be applied. Uncertainty in the ICER estimates will be presented as scatterplots on the cost-effectiveness plane and as cost-effectiveness acceptability curves.

Adverse events

Significant adverse events are defined as falls during exercise sessions, falls during the exercise period, musculoskeletal injuries, medical complications, hospital admissions and deaths. Significant adverse events during the exercise period are reported to the study administration.

Ethical considerations

The test battery consists of well-known and commonly used assessment tools in geriatric populations. Participants received an individual evaluation and adjustment of the programme to secure challenging but safe training. Each session was closely supervised by an experienced physiotherapist who also secured the participant by manual support if needed. The intervention is structured and targeted, but not fundamentally different from what could be given as part of normal clinical practice. Thus, the exercise programme is not regarded to be experimental or potentially harmful to the patients. Patients in the control group will receive today's best practice. The study has been approved by the regional ethics committee (REK 2010/3265-3).

Results

Inclusion was finalized in February 2013 with a total of 223 patients included, representing 90% of home-dwelling patients over the age of 70 years admitted for hip fracture within the catchment area during the inclusion period. Four months after the fracture, baseline registrations were performed on 183 (73%) participants, and 142 of the 250 eligible hip-fracture patients (57%) were randomized to the exercise intervention. The drop-out rate was 10% at T3 post-intervention and 15% at T4 1-year follow-up; in addition, eight participants died within the follow-up period. Twenty-seven of the 41 participants who were not randomized still met for assessments at T3 and 19 at 1-year follow-up (Figure 1).

Table 1 compare pre-fracture function and demographics for participants who were randomized to those who refused to participate in the exercise trial and those who were excluded or had died before baseline assessment. The results indicate that demographics and type of surgery did not differ between participants who were randomized or not, whereas those who refused to participate had poorer cognitive function, and those who were excluded on the basis of medical reasons or died had a reduced physical function prior to the fracture.

Discussion

This paper describes the protocol of a randomised controlled trial aimed to evaluate the effectiveness and cost effectiveness of a home-based exercise trial delivered at a time when regular rehabilitation is usually ended.

The described study can answer some of the limitations of previous studies. Strict inclusion criteria, especially on medical conditions and cognitive function in previous studies, have resulted in very low proportions of patients being considered eligible and included (Latham *et al.*, 2014; Orwig *et al.*, 2011). Further, hip-fracture patients participating in extended exercise programmes performed in an outpatient setting tend to have better cognitive function (Sylliaas *et al.*, 2011) than would be expected from prevalence studies on hip-fracture patients (Seitz *et al.*, 2011).

A strength of the present study is that patients are identified from daily screenings of operation lists and included already during the hospital stay. All hip fractures within the catchment area of the study are operated at the same hospital. Thus, this procedure ensures that the study sample is drawn from the population of interest, and the flow chart represents all potentially eligible hip-fracture patients within the catchment area.

A further strength is the collection of data on pre-fracture function shortly after the fracture and assessment of participants who are reluctant to participate in the exercise intervention that starts 4 months after

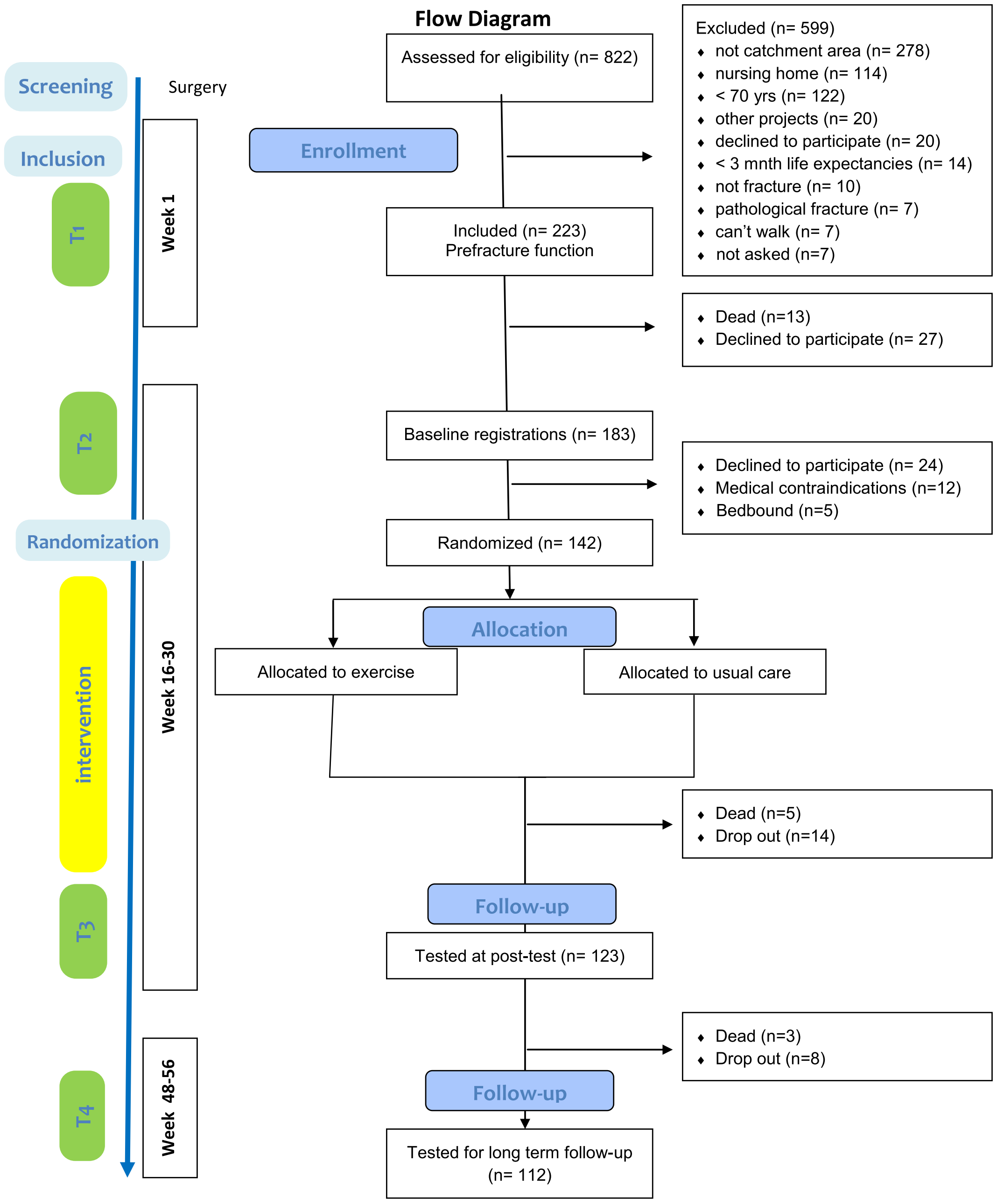

Figure 1. Study flow chart

inclusion but still willing to attend study assessments. Together, this provides a quite unique material for comparing characteristics of those who do participate and those who drop out during the follow-up period at different stages. Such knowledge is lacking and is of outmost importance when interpreting results of intervention trials in frail populations.

Inclusion criteria are broader than in most comparable trials. The excluded number of patients due to medical reasons are few (7%) taken into consideration the characteristics of the population and is, lower than in most comparable trials. The study has no exclusion criteria on cognitive impairment. Cognitive impairment is common within this population, and it is therefore of special interest to develop interventions that are effective in persons with impaired cognitive function. Still, results on pre-fracture function indicate that those who refuse to participate in the exercise

Table 1. Group differences in patient characteristics, pre-fracture function, fracture type and surgery between randomized participants and those who refused participation or were excluded or died before randomization

	Total (n = 223)	Randomized (n = 142)	Refusal (n = 50)	p	Dead or excluded (n = 31 (15+16))	p
Age (year) (mean (SD))	83.5 (6.2)	83.4 (6.2)	82.7 (6.0)	.828	85.2 (6.0)	.304
n (%)						
Women	161 (72)	110 (78)	32 (64)	.250	19 (61)	.205
Living alone	157 (70)	106 (75)	32 (64)	.362	19 (61)	.356
Hip fracture fall indoor	178 (80)	113 (72)	33 (70)	.253	26 (87)	.717
Walk aid/assistance indoor	59 (26)	32 (23)	10 (20)	.880	16 (52)	.016
Walk aid/assistance outdoor	104 (47)	63 (45)	19 (38)	.659	21 (68)	.054*
Intra-capsular fractures	131 (59)	82 (58)	32 (64)	.716	17 (55)	.954
Arthroplasty (proportion of intra-capsular)	114 (87)	66 (80)	25 (78)	.949	13 (76)	.949
Mean (SD)						
Barthel Index (0–20)	18.5 (2.1)	18.7 (2.0)	18.6 (2.0)	.976	17.7 (2.8)	.175
Nottingham extended activities of daily living (0–66)	42.1 (16.7)	45.1 (16.0)	39.2 (16.7)	.079	33.4 (16.4)	.002*
Clinical Dementia Rating Scale (0–18)	1.7 (3.2)	1.2 (2.5)	2.6 (3.9)	.051*	2.9 (4.0)	.058

*Significant group differences (One way Anova with Games Howell posthoc test assuming unequal variance).

programme have poorer cognitive function than those who have been randomized, despite that special care was taken to include this group, both in the design of the intervention and concerning study assessments procedures. These results underscore the need for more knowledge and targeted approaches towards these patients in future trials.

Few studies on exercise after hip fracture report the total number of patients who underwent surgery for a hip fracture during the inclusion period. Studies providing this information report inclusion rates between 14% and 44% (Orwig *et al.*, 2011; Sipila *et al.*, 2011; Ziden *et al.*, 2008a) suggesting that an inclusion rate of 57% 4 months after the surgery in the present study is relatively high.

There are good arguments for a more task specific approach to exercise after hip fractures and a lack of knowledge concerning the potentially beneficial effects of exercises on safety and effectiveness of gait after a hip fracture. In general, the effect of exercise interventions on physical performance such as muscle strength is better documented than the effect on more patient relevant outcomes (Auais *et al.*, 2012) underscoring that what persons can do is not the same as what they do. Assessment of gait characteristics beyond speed and extensive collection of data on physical activity by body-worn sensors provide new information on hip-fracture patients that may be important to evaluate the effect of the intervention on future fall risk and on everyday life function.

The study can also bring new information about cost effectiveness extending the rehabilitation period for hip-fracture patients. As standard rehabilitation in hip-fracture patients is usually finalized 4 months after surgery, an extended exercise programme such as this would represent new and extra costs. Cost effectiveness is therefore of special importance to assess but is so far lacking (Auais *et al.*, 2012).

Limitations of the study are the use of practice as usual for the control group instead of a sham intervention with comparable intervention time as for the exercise group. However, in this setting, it was regarded as important to be able to assess the gain of an extra effort compared with what these patients usually are offered. This is also relevant when calculating cost effectiveness of the programme. Another question is to which extent the results can be generalizable outside the municipality of Trondheim. Trondheim has a well-developed health-care and rehabilitation service, and it is expected that a substantial part of the participants in the control group will receive physiotherapy as part of usual care, which represents a danger of underestimating the effect a similar programme would have in another setting.

Implications for physiotherapy practice

This study can add new and important knowledge concerning the effectiveness and cost effectiveness of a physiotherapy-lead progressive gait and balance exercise programme for home-dwelling hip-fracture patients, intended to improve gait and activity. This may guide the development of more effective physiotherapy interventions for improving the outcome after hip fracture in frail, older patients and allow for a larger

proportion remain independent in their homes for a longer time.

Acknowledgements

We would like to thank the following physiotherapists who participated in the development and execution of the intervention: Bodil Bjåstad, Nina Nordvik, Silje Haslene, Gard Myhre, Synnøve Kirkeby-Garstad and Randi Granbo for her supervision on action research methods during the process of developing the intervention.

The research was supported by The Liaison Committee between the Central Norway Regional Health Authority (RHA) and the Norwegian University of Science and Technology (NTNU), the Municipality of Trondheim; and by the Norwegian Women's Health Association and the Norwegian Extra Foundation for Health and Rehabilitation through the EXTRA funds.

REFERENCES

Abellan van Kan G, Rolland Y, Andrieu S, Bauer J, Beauchet O, Bonnefoy M, Cesari M, Donini LM, Gillette Guyonnet S, Inzitari M, Nourhashemi F, Onder G, Ritz P, Salva A, Visser M, Vellas B. Gait speed at usual pace as a predictor of adverse outcomes in community-dwelling older people an International Academy on Nutrition and Aging (IANA) Task Force. The Journal of Nutrition, Health & Aging 2009; 13: 881–889.

Alley DE, Hicks GE, SHardell M, Hawkes W, Miller R, Craik RL, Mangione KK, Orwig D, Hochberg M, Resnick B, Magaziner J. Meaningful improvement in gait speed in hip fracture recovery. Journal of the American Geriatrics Society 2011; 59: 1650–1657.

Auais MA, Eilayyan O, Mayo NE. Extended exercise rehabilitation after hip fracture improves patients' physical function: a systematic review and meta-analysis. Physical Therapy 2012; 92: 1437–1451.

Bertram M, Norman R, Kemp L, Vos T. Review of the long-term disability associated with hip fractures. Injury Prevention 2011; 17: 365–370.

Chalder T, Berelowitz G, Pawlikowska T, Watts L, Wessely S, Wright D, Wallace EP. Development of a fatigue scale. Journal of Psychosomatic Research 1993; 37: 147–153.

Dolan P. Modeling valuations for EuroQol health states. Medical Care 1997; 35: 1095–1108.

Flay BR, Biglan A, Boruch RF, Castro FG, Gottfredson D, Kellam S, Moscicki EK, Schinke S, Valentine JC, Ji P. Standards of evidence: criteria for efficacy, effectiveness and dissemination. Prevention Science 2005; 6: 151–175.

Folden S, Tappen R. Factors influencing function and recovery following hip repair surgery. Orthopedic Nursing 2007; 26: 234–241.

Folstein MF, Folstein SE, Mchugh PR. "Mini-mental state". A practical method for grading the cognitive state of patients for the clinician. Journal of Psychiatric Research 1975; 12: 189–198.

Guralnik JM, Simonsick EM, Ferrucci L, Glynn RJ, Berkman LF, Blazer DG, Scherr PA, Wallace RB. A short physical performance battery assessing lower extremity function: association with self-reported disability and prediction of mortality and nursing home admission. Journal of Gerontology 1994; 49: M85–M94.

Handoll HH, Sherrington C, Mak JC. Interventions for improving mobility after hip fracture surgery in adults. Cochrane Database of Systematic Reviews 2011; CD001704.

Hauer KA, Kempen GI, Schwenk M, Yardley L, Beyer N, Todd C, Oster P, Zijlstra GA. Validity and sensitivity to change of the falls efficacy scales international to assess fear of falling in older adults with and without cognitive impairment. Gerontology 2011; 57: 462–472.

Helbostad JL, Taraldsen K, Granbo R, Yardley L, Todd CJ, Sletvold O. Validation of the Falls Efficacy Scale-International in fall-prone older persons. Age and Ageing 2010; 39: 259.

Holmes JD, House AO. Psychiatric illness in hip fracture. Age and Ageing 2000; 29: 537–546.

Hsu CL, Nagamatsu LS, Davis JC, Liu-Ambrose T. Examining the relationship between specific cognitive processes and falls risk in older adults: a systematic review. Osteoporosis International 2012; 23: 2409–2424.

Hughes CP, Berg L, Danziger WL, Coben LA, Martin RL. A new clinical scale for the staging of dementia. The British Journal of Psychiatry 1982; 140: 566–572.

Jellesmark A, Herling SF, Egerod I, Beyer N. Fear of falling and changed functional ability following hip fracture among community-dwelling elderly people: an explanatory sequential mixed method study. Disability and Rehabilitation 2012; 34: 2124–2131.

Kressig RW, Beauchet O, European GNG. Guidelines for clinical applications of spatio-temporal gait analysis in older adults. Aging Clinical and Experimental Research 2006; 18: 174–176.

Kwon S, Perera S, Pahor M, Katula JA, King AC, Groessl EJ, Studenski SA. What is a meaningful change in physical performance? Findings from a clinical trial in older adults (the LIFE-P study). The Journal of Nutrition, Health & Aging 2009; 13: 538–544.

Latham NK, Harris BA, Bean JF, Heeren T, Goodyear C, Zawacki S, Heislein DM, Mustafa J, Pardasaney P, Giorgetti M, Holt N, Goehring L, Jette AM. Effect of a home-based exercise program on functional recovery following rehabilitation after hip fracture: a randomized clinical trial. JAMA 2014; 311: 700–708.

Liu-Ambrose T, Nagamatsu LS, Hsu CL, Bolandzadeh N. Emerging concept: 'central benefit model' of exercise in falls prevention. British Journal of Sports Medicine 2013; 47: 115–117.

Lloyd BD, Williamson DA, Singh NA, Hansen RD, Diamond TH, Finnegan TP, Allen BJ, Grady JN, Stavrinos TM, Smith EU, Diwan AD, Fiatarone Singh MA. Recurrent and injurious falls in the year following hip fracture: a prospective study of incidence and risk factors from the sarcopenia and hip fracture study. The Journals of Gerontology. Series A, Biological Sciences and Medical Sciences 2009; 64: 599–609.

Mahoney FI, Barthel DW. Functional evaluation: the Barthel Index. Maryland State Medical Journal 1965; 14: 61–65.

Montero-Odasso M, Verghese J, Beauchet O, Hausdorff JM. Gait and cognition: a complementary approach to understanding brain function and the risk of falling. Journal of the American Geriatrics Society 2012; 60: 2127–2136.

Nouri FM, Nb L. An extended activities of daily living scale for stroke patients. Clinical Rehabilitation 1987; 1: 301–305.

Orwig DL, Hochberg M, Yu-Yahiro J, Resnick B, Hawkes WG, Shardell M, Hebel JR, Colvin P, Miller RR, Golden J, Zimmerman S, Magaziner J. Delivery and outcomes of a yearlong home exercise program after hip fracture: a randomized controlled trial. Archives of Internal Medicine 2011; 171: 323–331.

Perera S, Mody SH, Woodman RC, Studenski SA. Meaningful change and responsiveness in common physical performance measures in older adults. Journal of the American Geriatrics Society 2006; 54: 743–749.

Plummer-D'amato P, Kyvelidou A, Sternad D, Najafi B, Villalobos RM, Zurakowski D. Training dual-task walking in community-dwelling adults within 1 year of stroke: a protocol for a single-blind randomized controlled trial. BMC Neurology 2012; 12: 129.

Podsiadlo D, Richardson S. The timed "Up & Go": a test of basic functional mobility for frail elderly persons. Journal of the American Geriatrics Society 1991; 39: 142–148.

Rabin R, De Charro F. EQ-5D: a measure of health status from the EuroQol Group. Annals of Medicine 2001; 33: 337–343.

Resnick B, Galik E, Boltz M, Hawkes W, Shardell M, Orwig D, Magaziner J. Physical activity in the post-hip-fracture period. Journal of Aging and Physical Activity 2011; 19: 373–387.

Robinovitch SN, Feldman F, Yang Y, Schonnop R, Leung PM, Sarraf T, Sims-Gould J, Loughin M. Video capture of the circumstances of falls in elderly people residing in long-term care: an observational study. Lancet 2013; 381: 47–54.

Rohde G, Haugeberg G, Mengshoel AM, Moum T, Wahl AK. Two-year changes in quality of life in elderly patients with low-energy hip fractures. A case–control study. BMC Musculoskeletal Disorders 2010; 11: 226.

Rota V, Perucca L, Simone A, Tesio L. Walk ratio (step length/cadence) as a summary index of neuromotor control of gait: application to multiple sclerosis. International Journal of Rehabilitation Research 2011; 34: 265–269.

Schulz KF, Altman DG, Moher D. CONSORT 2010 statement: updated guidelines for reporting parallel group randomised trials. Journal of Pharmacology and Pharmacotherapeutics 2010; 1: 100–107.

Seitz DP, Adunuri N, Gill SS, Rochon PA. Prevalence of dementia and cognitive impairment among older adults with hip fractures. Journal of the American Medical Directors Association 2011; 12: 556–564.

Sheikh JI, Yesavage JA. Geriatric Depression Scale (GDS). Recent evidence and development of a shorter version. In: Brink TL (ed.). Clinical Gerontology: A Guide to Assessment and Intervention. NY: The Haworth Press, Inc., 1986.

Sherrington C, Henschke N. Why does exercise reduce falls in older people? Unrecognised contributions to motor control and cognition? British Journal of Sports Medicine 2013; 47: 730–731.

Singer JC, Prentice SD, McIlroy WE. Age-related changes in mediolateral dynamic stability control during volitional stepping. Gait Posture 2013; 38: 679–683.

Sipila S, Salpakoski A, Edgren J, Heinonen A, Kauppinen MA, Arkela-Kautiainen M, Sihvonen SE, Pesola M, Rantanen T, Kallinen M. Promoting mobility after hip fracture (ProMo): study protocol and selected baseline results of a year-long randomized controlled trial among community-dwelling older people. BMC Musculoskeletal Disorders 2011; 12: 277.

Sletvold O, Helbostad JL, Thingstad P, Taraldsen K, Prestmo A, Lamb SE, Aamodt A, Johnsen R, Magnussen J, Saltvedt I. Effect of in-hospital comprehensive geriatric assessment (CGA) in older people with hip fracture. The protocol of the Trondheim Hip Fracture trial. BMC Geriatr 2011; 11: 18.

Strobel C, Engedal K. MMSE-NR Norsk revidert Mini Mental Status Evaluation, 2008.

Sylliaas H, Brovold T, Wyller TB, Bergland A. Progressive strength training in older patients after hip fracture: a

randomised controlled trial. Age and Ageing 2011; 40: 221–227.

Sylliaas H, Brovold T, Wyller TB, Bergland A. Prolonged strength training in older patients after hip fracture: a randomised controlled trial. Age and Ageing 2012; 41: 206–212.

Taraldsen K, Vereijken B, Thingstad P, Sletvold O, Helbostad JL. Multiple days of monitoring are needed to obtain a reliable estimate of physical activity in hip fracture patients. Journal of Aging and Physical Activity 2014; 22: 173–177.

Vanswearingen JM, Perera S, Brach JS, Wert D, Studenski SA. Impact of exercise to improve gait efficiency on activity and participation in older adults with mobility limitations: a randomized controlled trial. Physical Therapy 2011; 91: 1740–1751.

Vickers AJ, Altman DG. Statistics notes: analysing controlled trials with baseline and follow up measurements. BMJ 2001; 323: 1123–1124.

Visschedijk J, Achterberg W, van Balen R, Hertogh C. Fear of falling after hip fracture: a systematic review of measurement instruments, prevalence, interventions, and related factors. Journal of the American Geriatrics Society 2010; 58: 1739–1748.

Visschedijk J, van Balen R, Hertogh C, Achterberg W. Fear of falling in patients with hip fractures: prevalence and related psychological factors. Journal of the American Medical Directors Association 2013; 14: 218–220.

Winter DA. Human balance and posture control during standing and walking. Gait & Posture 1995; 3: 193–214.

Yogev G, Plotnik M, Peretz C, Giladi N, Hausdorff JM. Gait asymmetry in patients with Parkinson's disease and elderly fallers: when does the bilateral coordination of gait require attention? Experimental Brain Research 2007; 177: 336–346.

Ziden L, Frandin K, Kreuter M. Home rehabilitation after hip fracture. A randomized controlled study on balance confidence, physical function and everyday activities. Clinical Rehabilitation 2008a; 22: 1019–1033.

Ziden L, Wenestam CG, Hansson-Scherman M. A life-breaking event: early experiences of the consequences of a hip fracture for elderly people. Clinical Rehabilitation 2008b; 22: 801–811.

Appendix A

	Equipment	Level 1	Level 2	Level 3	Level 4	Level 5
Walking 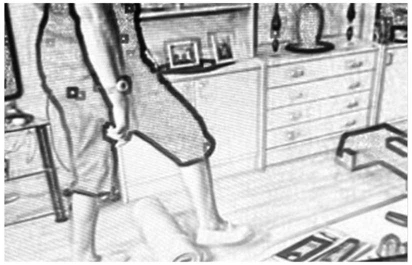	3- to 10-m walking path, no walking aid, supported by physiotherapist if necessary	Needs substantial support during walking: mostly forward walking at slow speed, short sessions	Needs moderate support while walking: simple instructions on increased step length/ walk the path in fewer steps and higher speeds.	Walks without support: Increased step length, speed variations, start/stop, turns. Simple dual task (e.g. talk while walking, counting and naming children) obstacle negotiation. Minimum of 10 minutes active walking	Variations in speed. Obstacle negotiation, narrow path walking and more challenging dual task. (calculating, birthdays of grandchildren, detailed road descriptions and shift keys from left to right pocket)	High speed, frequent, turns. Combinations of additional tasks. Outdoor walking in natural terrain or urban setting
Stepping 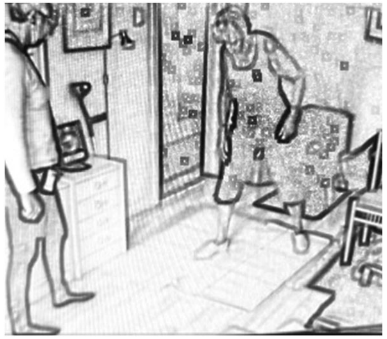	Anti-slip mat with a 3×3 grid pattern. (grid 40×40 cm) If necessary, supported by hand	Standing with support, weight transfer without stepping	With support: short steps forward and sideways	Without support, but with close supervision: steps in all directions in a predictable pattern. Slow speed. Minimum of 10 minutes active stepping	Without support: steps towards the edge of the mat, with higher speed than Level 3 and with variable patterns	High speed, unpredictable stepping patterns (instructed), weight vest

Step-Up 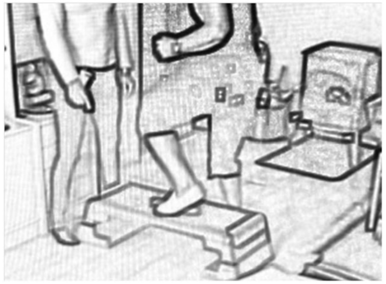	Adjustable step-box, weight vest	With support: tapping onto the step but not stepping up	With substantial support: able to do some of the four following exercises: 1) stepping up; 2) stepping up and over; 3) stepping sideways, one foot on each side of the step; and 4) lowering one foot at a time while standing on the step ('checking the water')	Little support, close supervision: exercises 1–4. Sessions of 3 × 15–20.	Without support: all four exercises. Series of 3 × 15–20	Increased height of the step-up box, weight vest. Higher speeds, longer sessions
Chair rise 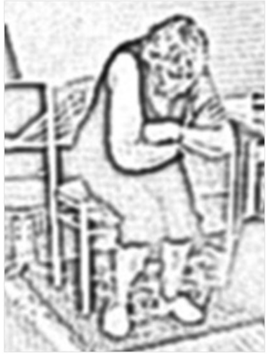	Chair 45–47 cm, pillows for adjusting height and weight vest	Raising/lowering on heightened chair with support	Raising/lowering on heightened chair without support	Raising/lowering on normal chair without support. 3 × 10–15 rep	Raising/lowering on normal chair with asymmetrical foot placements, and at higher speeds. Emphasis on controlled lowering	Raising/lowering on normal chair with weight vest
Lunge 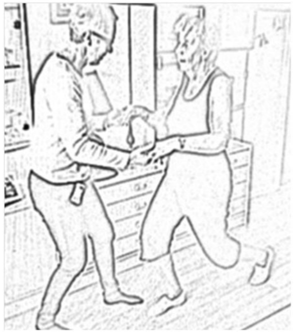	Weight vest	With substantial support: short forward lunges	With moderate support: short forward lunges	Without support: short forward lunges 3 × 8–12 rep	Lunges with 90° hip and knee flexion	Lunges with weight vest
